# Large-scale bioactivity analysis of the small-molecule assayed proteome

**DOI:** 10.1371/journal.pone.0171413

**Published:** 2017-02-08

**Authors:** Tyler William H. Backman, Daniel S. Evans, Thomas Girke

**Affiliations:** 1 Department of Bioengineering, University of California Riverside, Riverside, California, United States of America; 2 Institute for Integrative Genome Biology, University of California Riverside, Riverside, California, United States of America; 3 California Pacific Medical Center Research Institute, San Francisco, California, United States of America; Kyushu University, JAPAN

## Abstract

This study presents an analysis of the small molecule bioactivity profiles across large quantities of diverse protein families represented in PubChem BioAssay. We compared the bioactivity profiles of FDA approved drugs to non-FDA approved compounds, and report several distinct patterns characteristic of the approved drugs. We found that a large fraction of the previously reported higher target promiscuity among FDA approved compounds, compared to non-FDA approved bioactives, was frequently due to cross-reactivity within rather than across protein families. We identified 804 potentially novel protein target candidates for FDA approved drugs, as well as 901 potentially novel target candidates with active non-FDA approved compounds, but no FDA approved drugs with activity against these targets. We also identified 486348 potentially novel compounds active against the same targets as FDA approved drugs, as well as 153402 potentially novel compounds active against targets without active FDA approved drugs. By quantifying the agreement among replicated screens, we estimated that more than half of these novel outcomes are reproducible. Using biclustering, we identified many dense clusters of FDA approved drugs with enriched activity against a common set of protein targets. We also report the distribution of compound promiscuity using a Bayesian statistical model, and report the sensitivity and specificity of two common methods for identifying promiscuous compounds. Aggregator assays exhibited greater accuracy in identifying highly promiscuous compounds, while PAINS substructures were able to identify a much larger set of “middle range” promiscuous compounds. Additionally, we report a large number of promiscuous compounds not identified as aggregators or PAINS. In summary, the results of this study represent a rich reference for selecting novel drug and target protein candidates, as well as for eliminating candidate compounds with unselective activities.

## Introduction

High throughput screening (HTS) is a key technology for identifying bioactive small molecules for chemical genomics and drug discovery applications. Challenges encountered in the discovery of small molecules with high target specificity include experimental noise in HTS experiments and an extremely large search space. The potentially testable compound-protein target space consists of nearly two trillion possible combinations, if we regard each of the over 91 million small-molecules in the PubChem compounds database (at the time of writing) as potential drug candidates, and each of the annotated protein coding genes in the *Homo sapiens* genome (19950 genes according to GENCODE 25) as a potential drug target [1, 2]. This search space becomes much larger if we consider alternative splicing, non-protein biomolecule targets, and potential targets from other species, *e.g.* microbiome targets and parasite targets. In recent years, a substantial number of small molecule vs protein target assays have become available in the public domain, which investigate a portion of this search space. At the time of writing, the PubChem BioAssay database contains just over 230 million small molecule bioactivity outcomes, over half of which involve activity against a clearly defined protein target [3]. It includes most of the bioactivity data available in the public domain as it imports assays from many sources such as ChEMBL, and also provides negative (inactive) assay outcomes not reported in many databases [4]. This large data volume presents an opportunity to systematically investigate small molecule-target interactions, with the potential to provide insights relevant to future drug discovery efforts [3, 5–11]. These data also have potential utility for identifying and excluding drug candidates with undesirable binding properties (*e.g.* unselective promiscuous binders), developing multi-target (polypharmacological) drug treatments, predicting potential side and toxic effects of small molecules, and assessing the druggability of novel target proteins [8, 12–21]. The following gives a brief overview of previous work in this field.

Shortly after the NIH Molecular Library Roadmap Initiative made available large public screening data in PubChem BioAssay, Han *et al.* reported the distribution of assay participation, target selectivities, and target diversity in these data, while Zhang *et al.* later reported bias in target and compound selection among these data [8, 22, 23]. Hu and Bajorath quantified the distribution of active target proteins in the PubChem, DrugBank, and ChEMBL databases, and found that 37.4% of FDA approved drugs interact with more than five targets, while other active compounds tend to interact with only 1–2 targets, with only a 7.6% probability of more than five [4, 24, 25]. Recently, Jasial *et al.* analyzed compound promiscuity in PubChem BioAssay and found a median of 2 active targets for non-FDA approved compounds [26]. In comparison to previous work in this field, our study is unprecedented by providing a broader analysis of the publicly available small molecule bioactivity space, including target selectivity profiles within and across protein families considering variable evolutionary distances.

The concept of target selectivity has been introduced in previous literature, in order to quantify the number of distinct protein targets a compound exhibits activity against. Two common metrics for quantifying target selectivity have been frequently used. First, the total number of active targets across all participating assays, is referred to simply as target selectivity [8]. Second, the fraction of actives out of the total number of screened targets has been referred to as the hit ratio [27]. We analyzed target selectivity with both methods, as they have complementary strengths and weaknesses.

**Fig 1:**
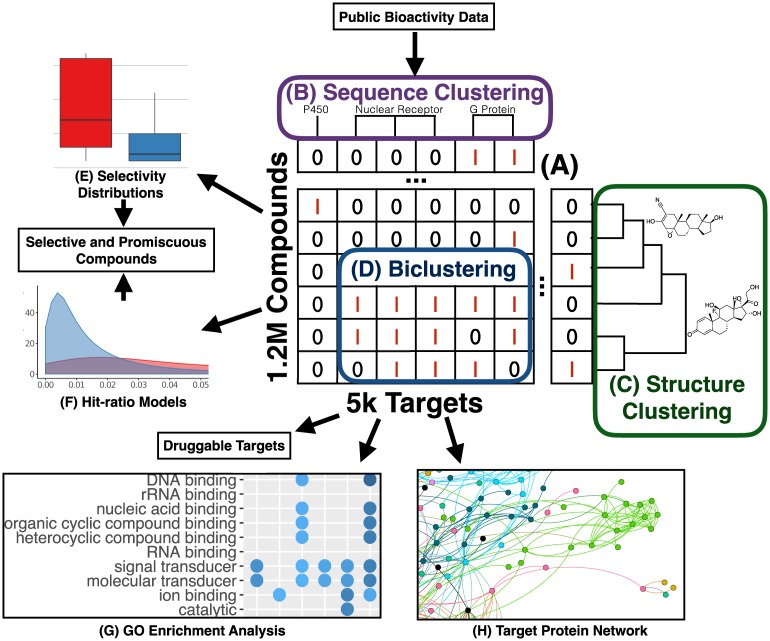
Bioactivity data mining strategy. Public bioactivity data was first summarized in a compound-target bioactivity matrix (**A**). Protein targets and small molecules were clustered by sequence (**B**) and structure (**C**) respectively, and compound-target sets with shared bioactivity profiles were identified with biclustering (**D**). For small molecules, the distributions of (**E**) target selectivity (the number of active targets) and (**F**) hit ratio (the fraction of screened targets that are active) were quantified. For protein targets, enriched GO (Gene Ontology) terms (**G**) among proteins with common bioactivity were identified, and a network (**H**) was constructed which connects target proteins with similar bioactivity profiles. These analyses highlight several interesting bioactivity patterns, identify promiscuous and selective compounds, and identify druggable protein targets and protein domains.

In this study, we mined large public bioactivity data to investigate many outstanding questions about the patterns of target selectivity among small molecules. Fig 1 provides a visual overview of important steps in our data analysis strategy. To investigate why FDA approved drugs on average exhibit activity against a greater number of targets than non-FDA compounds, we computed the target selectivity of small molecules against protein clusters obtained with three distinct methods that classify protein sequences across increasingly large evolutionary distances. While FDA approved drugs have on average a greater number of targets, these targets more frequently share sequence similarity than targets of non-FDA active compounds.

We also found that many of these multi-target FDA approved drugs fall into biclusters, where a common set of drugs share activity against a common set of protein targets that are enriched for common molecular function annotations, suggesting a shared chemical mechanism leading to cross-reactivity. To determine which targets are more accessible to small molecule perturbations than others, we quantified the number of active compounds for targets grouped by shared protein domains, and found active compounds for targets which contain 32.4% of the domains present in the *H. sapiens* proteome. Clustering the targets by similar amino acid sequences, we found 9120 active target outcomes for FDA approved drugs not currently annotated in drug databases. By quantifying the rate of agreement among millions of replicated compound-target pairs across distinct assays, we estimate that over half of these novel results are accurate bioactivity outcomes. To investigate the frequency of highly promiscuous compounds, we used a statistical model to infer the hit ratio of each compound, and report 1157 likely-promiscuous compounds not previously identified by two common methods of identifying promiscuous compounds, aggregator assays and PAINS substructures [12, 28].

## Results and discussion

### Bioactivity data

#### Bioactivity data curation and overview

The bioactivity data analyzed by this study were downloaded from PubChem BioAssay on April 6th, 2016. They included 1.2 million distinct small molecule structures tested against 5069 protein targets in 68029 assay experiments [3]. We were able to utilize all experiments annotated with a single clearly defined protein target, and reporting an active score for at least one small molecule. Assays with no active scores, or no machine readable protein target annotation were excluded. Much of this data summarizes the results from primary screening experiments which provide only binary active/inactive results, but we also analyze confirmatory assays, if binary calls are also provided.

As compounds were screened against variable numbers of targets, the compound vs target bioactivity space obtained from PubChem BioAsssay is sparse. Currently, there are 162 million compound-target activity records available, populating 2.6% of the full bioassay matrix with at least one measurement. Within the explored bioactivity space, active values are relatively rare (just over 2.3 million), representing just over 1.3% of total tested values, or about 0.027% of the total space. If we consider just the 566983 “highly screened” compounds tested against at least 10 distinct targets, the density of tested bioactivity outcomes increases to 6.1%. The patterns of bioactivity among these “highly screened” compounds are the focus of this study, as they provide information about bioactivity profiles across many targets. Collapsing the protein target space by merging very similar sequences, such as truncations, and close orthologues and paralogues, reduces these targets from 5069 protein targets to 2249 target clusters, producing a smaller and more dense bioactivity matrix. This is described in more detail in the Methods section (see “Clustering Protein Targets by Sequence”). A subset of the bioactivity space is non-sparse, with a set of 81660 compounds by 247 target clusters that has been explored 100%, which we discuss in S1 Text and provide as a downloadable reference for users in S7 File.

To facilitate comparisons throughout this study among FDA approved and all other compounds, we obtained a list of the 1173 FDA approved drugs with known PubChem ids from the DrugBank database, and quantified the number of screened targets for both categories [25]. Table 1 shows the distribution of total screened protein targets for the compounds in PubChem BioAssay. The overall distribution is also plotted in S3 Fig in Supporting Information. While a disproportionately large fraction of non-FDA compounds were screened against a small number of targets, the distribution of screening frequencies between highly screened FDA approved and non-FDA compounds is similar. Highly screened FDA approved drugs were screened against a mean of 242 targets (median 184), while highly screened non-FDA approved compounds were screened against a mean of 224 (median 280) targets. Therefore, these data allow us to compare patterns of target selectivity between many FDA approved and non-FDA approved compounds with similar assay participation profiles. Additionally, the hit ratio statistical model we introduce below in the “Promiscuous Binders and Hit Ratio Statistical Model” section provides a robust method of comparing target selectivity across compound sets with varying assay participation.

**Table 1:** Screening frequency of FDA approved and non-FDA compounds against increasing numbers of protein targets. Data is included from all assay experiments in PubChem BioAssay annotated with one clearly defined protein target, and reporting an active score for at least one small molecule. Multiple assays against the same target are counted only once.

Screened Protein Targets	FDA Approved Drugs	Non-FDA Compounds
1	31	359135
2–4	41	135786
5–9	27	151385
10–49	197	150202
50–99	128	51849
100–199	150	30277
200–299	85	69098
300–399	94	202225
400–499	106	63219
≥500	139	82

When comparing the bioactivity profile and target selectivity among compounds, we focus on compounds with evidence of activity against at least one protein target, as active compounds are more likely to be of biological or pharmacological interest. Of the 566983 “highly screened” compounds mentioned above, 312308 have also been found active against one or more targets. Among the highly screened active compounds, 759 are FDA approved drugs, whose patterns of target selectivity we compare and contrast with non-FDA approved highly screened active compounds.

#### Data quality and reproducibility

Systematically analyzing public bioactivity data presents many data quality challenges stemming from experimental error, and missing or incorrect annotation. While efforts such as the BioAssay Research Database (BARD) and BioAssay Ontology are underway to curate a set of assays with detailed high quality annotations, these represent a very small subset of the publicly available bioactivity data [29, 30]. To assess the reliability of the data, we estimate an error rate for compound-target combinations tested multiple times in separate assays by quantifying how often the results agree or disagree. This estimate combines all errors causing *in vitro* screening outcomes from different primary screening assays to disagree, such as underlying experimental noise, data curation and annotation errors, as well as disagreement resulting from unique experimental context or conditions for a particular assay, that are not provided in a machine readable format. While we can quantify how often activity outcomes disagree across different assays, the PubChem BioAssay data does not include information about the exact cause of a disagreeing activity outcome, or in which assay the error occurred in. As this data includes assays of variable design and robustness, individual assays will have different error rates. However, our estimate represents the probability of any individual compound-target activity outcome reporting an incorrect result when combined and analyzed in aggregate, as we do in this study.

Table 2 shows the number of distinct compound-target pairs that were screened a given number of times. In S1 Text we solve algebraically for the error rate based on the frequency of agreeing or disagreeing sets among compound-target pairs tested in exactly two different assays. As explained and justified in S1 Text, our estimate is an approximation which requires two simplifying assumptions. First, we set the average false positive and false negative rates across the entire dataset equal, and estimate an overall error rate *e*. Second, we assume that the fraction of true active compound-target pairs in the total PubChem BioAssay data is approximately the same for both the set with two replicates, and the larger set of data with more or less than two replicates. Based on these assumptions, we estimate an error rate of approximately 0.698%, representing the probability of any individual bioactivity outcome reporting the opposite of its true result.

**Table 2:** Screening Frequency. The number of distinct compound-target combinations screened in multiple assays, listed for increasing numbers of assays.

Times Screened	Number of Compound-Target Pairs
2	21220270
3	3308744
4	726700
5	29610
≥6	376787

The high throughput screening experiments we analyze here require choosing a hit threshold, which assigns a binary active or inactive outcome to each compound tested, based on the magnitude of its experimentally measured activity level. The specific hit threshold is a subjective choice of the experimentalist that balances the acceptability of false positives and false negatives for a given purpose, and is not provided to us in a machine readable manner. Thus, it is not feasible to provide here a precise estimate of the fraction of actives which are true positives [31]. In many drug discovery efforts, false positives are more problematic than false negatives. As a result, experimentalists are more likely to choose a stringent activity cutoff, biased towards avoiding false positives. As such, these data suggest a rough estimate on the lower bound of the fraction of active compound-target outcomes which are true positives of approximately 66%, but it may be higher. Therefore, we expect that despite a considerable error rate, more than half of the unreplicated positive activity outcomes in these data are meaningful in the context they are used for in this study.

#### Protein target diversity

To assess the target protein diversity represented in PubChem BioAssay, we enumerated the number of distinct targets by three methods which group targets across increasingly large evolutionary distances, including (i) unique protein identifiers, as well as clustering (ii) by protein sequence similarity and (iii) by Pfam domains [32]. The 68029 assay experiments we analyzed grouped into 5069 clusters of assays sharing an identical distinct GenBank Protein GI (Gene Identifier), each of which has a unique amino acid sequence [33]. By clustering these targets together based on an amino acid sequence identity of at least 60%, a kClust E-value ≤ 10^−4^, and an alignment coverage of at least 80% for the longer sequence, we identified 2249 distinct target clusters [34]. This method clusters together minor truncations engineered for screening purposes, as well as closely related orthologues and paralogues (see “Clustering Protein Targets by Sequence” in Methods). To investigate target diversity at the domain level, we mapped Pfam-A domains to each protein target with a distinct GI as described in the Methods section under “Protein Annotations and GO Enrichment”. The domain mapping results were used for two different domain-based protein clustering approaches: *single domain clustering* and *domain composition clustering*. The former assigns proteins to clusters based on the presence of single domains. This approach assigns multiple domain proteins to several clusters (*i.e.* “fuzzy clustering”). To investigate the magnitude of the effect these duplications may have on downstream analyses, we also performed *domain composition clustering*, where multiple domain proteins are assigned to the same cluster if they share the same domains disregarding their order. This stricter method results in nearly unique cluster assignments of proteins while enabling the identification of domains occurring on the same targets. Because we were mainly interested in assessing which Pfam domains participated in bioassays rather than individual combinations of domains, most downstream analyses involving Pfam domains were performed with the *single domain clustering* results. It is also important to point out that the exact binding site within the bioassayed proteins is not readily available to us. This means it is not possible to determine for most bioassayed proteins whether their annotated Pfam domain(s) overlap with the actual binding site of the corresponding compounds. Thus, compound-target interactions investigated by this study refer to entire proteins rather than specific Pfam domains. Additional annotation data or experimental inquiry would be necessary to pinpoint the true target domain(s) within proteins.

**Fig 2:**
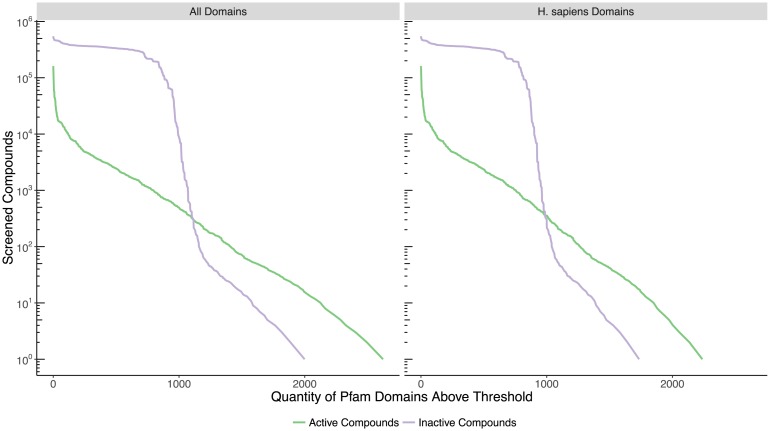
Pfam domain screening participation. The quantity of Pfam domains is plotted on the horizontal (x) axis whose protein targets have at least as many active or inactive compounds as shown on the vertical (y) axis. The left panel includes all Pfam domains in the PubChem BioAssay targets, while the right panel includes just those domains also present in the *H. sapiens* proteome, including non-*H. sapiens* targets which share a common domain with an *H. sapiens* protein.

To assess the Pfam domain participation in the bioassay data, we quantified the distribution of screening participation by active and inactive compounds for the targets with different Pfam domains in Fig 2, with all domains shown in the left panel, and the subset present in the *H. sapiens* proteome shown in the right panel. In total there are 2838 distinct Pfam domains represented in PubChem BioAssay that are associated with active compounds, and therefore have at least some evidence of druggability. In comparison, 32.4% of the 7431 distinct Pfam domains represented in the *H. sapiens* proteome are also represented in these bioassays and report active compounds, while 27.1% of them were screened directly on *H. sapiens* proteins. Additionally, there are proteins with active compounds in PubChem BioAssay which contain 433 Pfam domains not present in the *H. sapiens* proteome, many of which are domains restricted to bacteria and plants. There are 795 Pfam domains that are extremely highly screened, with activity outcomes for over 200k compounds each. The number of domains with active compounds is greater than inactive compounds, due to assays which do not report inactive outcomes. While these are mostly small assays reporting few activity outcomes, they substantially increase the information about the druggable space by reporting active compounds for 792 domains not present in the other assays, 614 of which are present in the human proteome. Because proteins often contain multiple Pfam domains, duplications are unavoidable with this type of protein family clustering. Out of 5069 distinct protein targets, 79% (3989 proteins) have two or more domains. We clustered these 3989 multi-domain proteins based on the composition of Pfam domains in each (*domain composition clustering*), and found only 1959 distinct clusters with a unique combination of domains, showing that the same sets of multiple-domains frequently occur together. As a resource to readers, we provide the domain composition clusters for each domain in S8 File of Supporting Information. We also use this result to show in most tables and figures only one representative domain for a set co-occurring together on the same targets. The details of this approach are described in the Methods section (see “De-duplication of Single Domain Clusters”).

**Fig 3:**
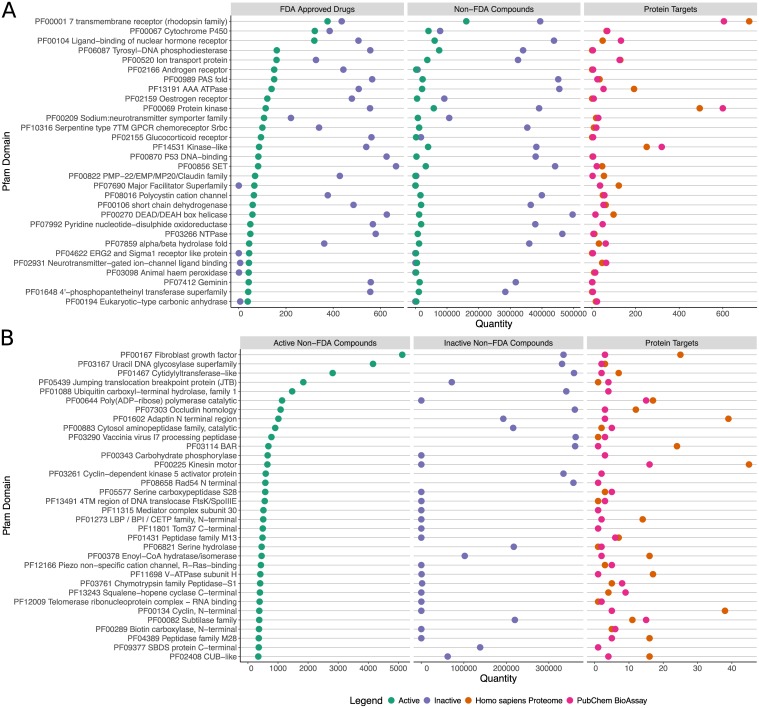
Frequency of active pubchem bioassay compounds across protein target domains. The target proteins represented in PubChem BioAssay have been classified by Pfam protein domains present in the *H. sapiens* proteome (vertical axis). We report data for all proteins which encode a Pfam domain present in the *H. sapiens* proteome, even if the assay was performed against a protein from another species. We show here only domains with at least 100 amino acid residues in the homology model, to avoid small repeats and domains unlikely to be drug targets. Additionally, we report for multi-domain clusters only the most frequent and functionally descriptive members as outlined in the Methods section (see “De-duplication of Single Domain Clusters”). Domains of unknown function (DUFs) were also removed since they are rarely the functional target of bioassays. The quantity of targets with each domain among the PubChem BioAssay data, and within the *H. sapiens* proteome (all proteins, including those not screened in PubChem BioAssay) are shown on the right in both plots. **(A)** The top 30 Pfam domains with the greatest number of active FDA approved drugs, in decreasing order. **(B)** The top 34 Pfam domains with the greatest number of non-FDA compounds, but no active FDA approved drugs, in decreasing order. A full table is provided in the S2 File of Supporting Information including the number of active compounds for each domain, non-*H. sapiens* domains, all domains occurring on the same proteins, and domains with less than 100 residues.

Fig 3A enumerates the relative abundance of active FDA approved drugs, active non-FDA compounds, and total protein targets for the 30 domains with the largest number of active FDA approved drugs. As can be seen by comparing column 1 (FDA Approved Drugs) to column 2 (Non-FDA Compounds), the fraction of screened compounds active against each domain is significantly higher for FDA approved drugs. However, due to the much greater number of non-FDA compounds, the total number of non-FDA actives is much higher than the number of FDA approved drugs. For comparison, the number of proteins within each Pfam domain is given in Column 3 of Fig 3A for both proteins represented in PubChem BioAssay and those encoded in the *H. sapiens* genome. In some cases, the number of targets in PubChem BioAssay exceeds those in the *H. sapiens* proteome because it includes targets from other species, as well as engineered targets (*e.g.* truncations) developed for screening purposes. The proteins targeted by the greatest number of FDA approved drugs include rhodopsin-like GPCRs, cytochrome P450 enzymes, and nuclear hormone receptors, with a large number of non-FDA compounds also targeting these proteins.

Several domains such as Tyrosyl-DNA phosphodiesterase and protein kinase have a large number of active non-FDA compounds compared to the ordering of domains, which is based on a decreasing number of FDA approved active compounds. In cases such as kinases this may be explained by the large number of assays screening distinct targets within a large protein family. However, in other cases such as Tyrosyl-DNA phosphodiesterases there are a comparatively large number of active non-FDA compounds despite relatively fewer target proteins screened within this much smaller family. Overall, we found 486348 non-FDA compounds active against individual targets (distinct GenBank GIs) that also have active FDA approved drugs, representing a set of potentially novel compounds active against potentially therapeutic targets.

As we reported previously, the FDA approved drugs show activity against 1789 protein targets, whereas the non-drug compounds show activity against an additional 3020 protein targets, of which 901 are substantially distinct at the sequence level, based on the clustering by sequence similarity mentioned above [35]. Fig 3B lists the top 34 domains contained in proteins with no active FDA approved compounds, but the greatest number of non-FDA compounds. These targets with domains not known to be accessible to FDA approved drugs represent a greatly expanded space of potentially druggable targets and small molecule drug candidates. In total, we found 153402 compounds active against individual targets (distinct GenBank GIs) with no active FDA approved drugs. While some of these compounds will be false positives due to experimental noise, the magnitude of actives suggests a large quantity of truly active compounds. We provide the number of active FDA approved and non-FDA compounds for the full set of Pfam domains in the S2 File of Supporting Information.

### Target selectivity

#### Target selectivity distribution

**Fig 4:**
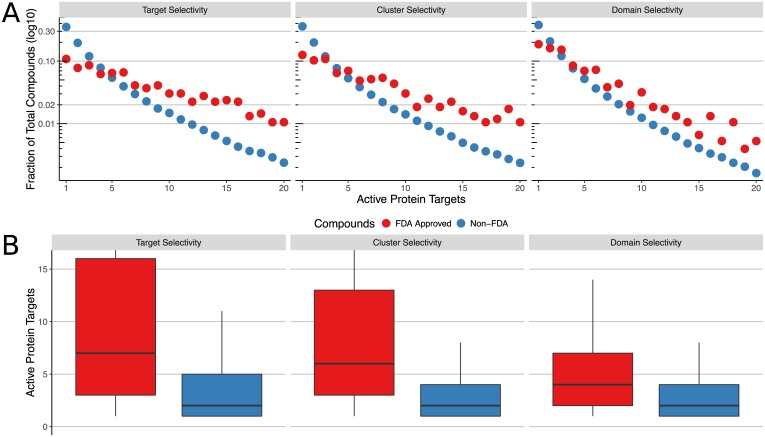
Distribution of active proteins per compound. Both plots show the distribution of target selectivity among PubChem BioAssay compounds, with selectivity quantified by three methods which cluster protein targets across increasingly large evolutionary distances, as described in the text. FDA approved drugs (red) are shown separately from non-FDA approved compounds (blue). **(A)** Semi-log plot of the target selectivity distributions, where horizontal (x) axis represents the number of active protein targets and/or protein target clusters, while the vertical (y) axis represents the fraction of each compound set that is active against a particular number of targets. **(B)** Box plot of the target selectivity distributions, with horizontal lines at the 25%, 50%, and 75% quantiles for each distribution. The vertical (y) axis represents the number of active protein targets and/or protein target clusters. Whiskers extend to 1.5 times the inter-quartile range, however we limit the vertical (y) axis to 16 in order to zoom into the higher density region.

Highly screened bioactive small molecules can be categorized according to target selectivity, which is the number of distinct protein targets they show activity against. By quantifying the distribution of target selectivities, we can identify highly selective and less selective compounds, as well as compare the selectivities of FDA approved drugs to non-FDA approved compounds. To address this, we computed the distribution of target selectivities among the highly screened active compounds in PubChem BioAssay, each of which were tested against 10 or more protein targets, and active against at least one. We computed target selectivity based on the three types of protein clustering methods mentioned in the previous section. “Target selectivity” counts each target with a distinct amino acid sequence (distinct GenBank Protein Gene Identifiers) separately, while “cluster selectivity” counts the number of sequence-based clusters a compound shows activity against. Third, “domain selectivity” counts activity against any set of targets sharing a common Pfam protein domain only once. Due to the existence of protein targets with multiple domains, we compute the number of domain clusters independently for each compound. For example, if a compound is active against 5 targets, but 4 share a common domain, its domain selectivity is 2. This is the same as counting the number of connected components in a graph where each node represents an active protein target, and edges represent target pairs sharing a common Pfam domain. The distribution of counts for all three clustering methods is shown in Fig 4. Fig 4B includes a boxplot which highlights the quantiles for each distribution, while in Fig 4A, counts are shown for values up to 20. There are an additional 144 FDA approved drugs and 6285 non-FDA compounds with greater than 20 individual active targets. These are not shown in Fig 4A as they represent a very small fraction of the total compounds, and for highly promiscuous compounds, may simply represent the number of screened targets instead of a biologically relevant quantity. The “Promiscuous Binders and Hit Ratio Statistical Model” section below quantifies and visualizes selectivity in a way that normalizes by assay participation, allowing us to investigate selectivity distributions among highly promiscuous compounds.

Interestingly, the FDA approved drugs show a much greater frequency of activity against many targets, and reduced frequency of activity against only one or two targets as compared to non-FDA compounds, as shown in Fig 4. In S1 Text of the Supporting Information we also provide a table with median, mean, and trimmed mean values for all three clustering methods. We performed a one sided Mann-Whitney-Wilcoxon test to determine if the FDA approved drugs have higher counts than the non-FDA approved compounds vs. the null hypothesis that they have equal or lower counts. The values W were 180M, 173M, and 154M for target, cluster, and domain selectivity counts respectively, with p-value < 2.2 * 10^−16^ by normal approximation for all three comparisons. While this observation was reported in previous literature, we report an even higher number of targets for the FDA approved compounds, based on the larger volume of data we analyze here [24, 36]. This higher number of active targets is unlikely to be due to biased assay participation, because as discussed in the above “Bioactivity Data Curation and Overview” section, non-FDA compounds were screened against a higher median number of targets, yet show a lower median number of active targets. Additionally, in the “Promiscuous Binders and Hit Ratio Statistical Model” section below, we report that this trend is still present when analyzed with a statistical model that accounts for the individual assay participation of each compound.

As shown in Fig 4, the target promiscuity of FDA approved drugs decreases substantially as related targets are clustered across increasingly large evolutionary distances, while the target promiscuity of non-FDA compounds decreases to a much lesser extent. For the FDA approved drugs, the median selectivity drops from 7 to 4 when targets sharing common domains are clustered. The Mann-Whitney-Wilcoxon test results in the previous paragraph also quantify the magnitude of this difference. As this test is based on position in a ranked list, the result indicates that for a large number of FDA approved compounds, there is a greater number of non-FDA compounds with higher domain selectivity counts, than the number of non-FDA compounds with higher target selectivity counts. These results highlight a fundamental difference in the overall trend of bioactivity between FDA and non-FDA compounds active against many targets. While the FDA approved drugs tend to be active against many more targets than non-FDA compounds, a greater fraction of these targets share common Pfam domains and/or overall sequence similarity. While a substantial fraction of the active targets of FDA approved drugs are closely related, the FDA approved drugs also exhibit activity against a slightly higher number of unrelated targets than the non-FDA compounds.

To determine whether the greater promiscuity of FDA approved drugs for related targets is caused by a biased screening participation within certain target families, we performed comparisons where we normalized the differences in assay participation among FDA and non-FDA drugs. Due to the sparsity of the data, this analysis had to be restricted to a subset of target clusters with sufficient bioassay data where it was possible to obtain equal assay participation among FDA and non-FDA drugs by iteratively and randomly removing bioassay outcomes from the more frequently tested cases. A detailed outline this analysis is provided in the S1 Text in Supporting Information (see “Target Selectivity Distribution Among Targets Sharing a Common Pfam Domain”). This result demonstrates that the greater fraction of active targets sharing common Pfam domains among the FDA approved compounds is still observable after controlling for differences in assay participation at the individual domain level. One plausible explanation for this trend is that many FDA approved drugs have been optimized to bind to their targets with high affinity resulting in more frequent cross-reactions with related targets than less optimized non-FDA drugs.

We further explore the selectivity against distinct Pfam domain families in the next section. As a resource for readers, we report the target selectivity, cluster selectivity, and domain selectivity for all highly screened actives in the S1 File of Supporting Information.

#### Selectivity across pfam domains

As the FDA approved drugs exhibit wide variation in target selectivity, with both highly selective, and highly promiscuous compounds, we wanted to determine whether promiscuous and selective compounds exhibit activity against different subsets of the protein target space. To answer this question, we identified the highly screened compounds exhibiting activity against the target proteins grouped by Pfam domains. We then computed for each domain the median domain selectivity counts of the active compounds. Domain selectivity is the same as introduced in the “Target Selectivity Distribution” section above, where active targets sharing a common domain are counted only once. We performed this separately for the FDA approved, and non-FDA compounds, while excluding domains with less than 10 active compounds from both sets. Table 3 quantifies the number of domains grouped into 8 bins of median domain selectivity, showing an extremely wide variation of median domain selectivities, including both domains whose active compounds tend to be highly promiscuous, and domains whose active compounds tend to be highly selective. Table 4 lists the top 16 Pfam domain families whose active FDA approved drugs are most promiscuous across proteins with different domains, while Table 5 lists the top 19 Pfam domain families whose active FDA approved drugs are most selective across proteins with different domains. Domains not present in the *H. sapiens* proteome are not shown in Tables 4 and 5, but were included in the analysis and are available in the S2 File of Supporting Information.

**Table 3:** Frequency of pfam domains binned by median domain selectivity of active compounds. Each row represents a set of Pfam domains whose active compounds (against targets with that domain) have a median domain selectivity in the range specified. Domain selectivity is the same as introduced in the “Target Selectivity Distribution” section above, where active targets sharing a common domain are counted only once. The ranges are ordered from top to bottom by increasing number of distinct domain active targets. We report bin counts separately for FDA Approved and Non-FDA compounds.

Bins of Median Domain Selectivity	FDA Approved Domain Counts	Non-FDA Domain Counts
2–4	6	113
5–7.5	80	169
8–10.5	119	81
11–13.5	71	16
14–16.5	63	7
17–19.5	29	3
20–22	17	2
23.5–25.5	6	0

**Table 4:** Top 16 pfam domains with least selective active drugs. Only domains present in the *H. sapiens* proteome are shown, and are sorted and selected by decreasing domain selectivity among FDA approved drugs. We also excluded domains with under 100 amino acid residues in the homology model, to avoid small repeats and domains unlikely to be drug targets. Additionally, we report for multi-domain clusters only the most frequent and functionally descriptive members as outlined in the Methods section (see “De-duplication of Single Domain Clusters”), and also removed domains of unknown function (DUFs). To the left of the slash in each column is the median target selectivity of FDA approved compounds active against this domain, and to the right is the median selectivity of non-FDA compounds. Selectivity is quantified by three methods of increasingly grouped protein targets as described in the text. For example, the non-FDA approved compounds active against targets with the PF00183 domain are active against a median of 12 targets with distinct GenBank identifiers, so this domain has a 12 after the slash in the first column.

Domain	Median Target Selectivity FDA Approved/non-FDA	Median Cluster Selectivity FDA Approved/non-FDA	Median Domain Selectivity FDA Approved/non-FDA
PF16178 Dimerisation domain of Ca+-activated chloride-channel, anoctamin	41/8	36/7	25/6
PF03520 KCNQ voltage-gated potassium channel	44.5/7	40/7	25/6
PF10488 Phosphatase-1 catalytic subunit binding region	49/14	46.5/14	23.5/12
PF10401 Interferon-regulatory factor 3	46.5/8	45.5/8	22/7.5
PF09038 Tumour suppressor p53-binding protein-1 Tudor	43.5/6	36.5/6	21/6
PF02518 Histidine kinase-, DNA gyrase B-, and HSP90-like ATPase	36/12	31/12	21/10
PF00180 Isocitrate/isopropylmalate dehydrogenase	33/11	27/10	19/9
PF02127 Aminopeptidase I zinc metalloprotease (M18)	31/9	29/9	19/8
PF00758 Erythropoietin/thrombopoietin	41/10	28/9	18.5/7
PF00044 Glyceraldehyde 3-phosphate dehydrogenase, NAD binding domain	33/13	27/13	18/12
PF00011 Hsp20/alpha crystallin family	29/10	28/10	18/5
PF01231 Indoleamine 2,3-dioxygenase	41.5/13	31/12.5	18/9
PF00452 Apoptosis regulator proteins, Bcl-2 family	29/10	27/10	17.5/9
PF00817 impB/mucB/samB family	23/11	23/11	17/9
PF03388 Legume-like lectin family	19/6	19/6	17/5
PF00443 Ubiquitin carboxyl-terminal hydrolase	23/19	23/18	17/15

**Table 5:** Top 19 pfam domains with most selective active drugs. Only domains present in the *H. sapiens* proteome are shown, and are sorted and selected by increasing domain selectivity among FDA approved drugs. We also excluded domains with under 100 amino acid residues in the homology model, to avoid small repeats and domains unlikely to be drug targets. Additionally, we report for multi-domain clusters only the most frequent and functionally descriptive members as outlined in the Methods section (see “De-duplication of Single Domain Clusters”), and also removed domains of unknown function (DUFs). To the left of the slash in each column is the median target selectivity of FDA approved compounds active against this domain, and to the right is the median selectivity of non-FDA compounds. Selectivity is quantified by three methods of increasingly grouped protein targets as described in the text. For example, the non-FDA approved compounds active against targets with the PF00144 domain are active against a median of 6 targets with distinct GenBank identifiers, so this domain has a 6 after the slash in the first column.

Domain	Median Target Selectivity FDA Approved/non-FDA	Median Cluster Selectivity FDA Approved/non-FDA	Median Domain Selectivity FDA Approved/non-FDA
PF06512 Sodium ion transport-associated	7/4	5.5/4	3/2.5
PF00144 Beta-lactamase	11.5/6	5.5/6	4/5
PF14580 Leucine-rich repeat	14/6	12/6	4/5
PF00324 Amino acid permease	14/7	7/7	5/6
PF00194 Eukaryotic-type carbonic anhydrase	18/11	12/6	5/2
PF02931 Neurotransmitter-gated ion-channel ligand binding domain	13/5	10/4	5.5/2
PF00001 7 transmembrane receptor (rhodopsin family)	13/4	10/4	6/3
PF13481 AAA domain	15/9	12.5/9	6/8
PF09818 Predicted ATPase of the ABC class	15/6	12.5/6	6/5
PF07859 alpha/beta hydrolase fold	11/4	9/4	6/4
PF00186 Dihydrofolate reductase	9/6.5	8.5/6	6/3.5
PF01494 FAD binding domain	13/13	10/13	6/11
PF02155 Glucocorticoid receptor	13.5/7	12/7	6/3
PF00104 Ligand-binding domain of nuclear hormone receptor	13/6	10/6	6/5
PF00109 Beta-ketoacyl synthase, N-terminal domain	8.5/4	8/4	6/4
PF00061 Lipocalin / cytosolic fatty-acid binding protein family	12/16	8/14.5	6/9.5
PF02159 Oestrogen receptor	14/6	12/6	6/4
PF00067 Cytochrome P450	14/4	11/3	6/3
PF00075 RNase H	12.5/8	10.5/8	6/7

**Fig 5:**
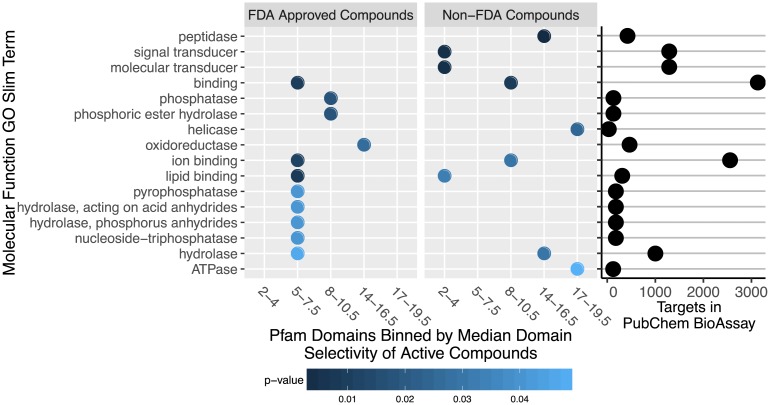
Molecular Function Gene Ontology (MF GO) slim term enrichment vs domain selectivity. Pfam domains are binned by the median domain selectivity of active compounds against targets with these domains, as in Table 3. The domains in each bin were computed separately based on FDA approved and non-FDA compounds, shown here side by side. For each bin of domain selectivity, the enrichment of MF GO slim terms against the background of all bins is shown. Enriched terms are sorted increasingly by the lowest p-value obtained, with all terms shown here having a p-value < 0.05. The right column dot plot shows the number of protein targets in PubChem BioAssay annotated with each MF GO slim term.

To determine whether functional activities are enriched within the individual selectivity bins of Table 3, we used the Molecular Function Gene Ontology annotations (MF GO) of the corresponding Pfam domains to perform a GO term enrichment test based on the hypergeometric distribution (see “Protein Annotations and GO Enrichment” Methods section) [37]. Since we were mostly interested in enrichments within general functional categories, we restricted this analysis to the slim terms of the MF GO. This allows us to identify functional categories that are more abundant within the selectivity bins than one would expect by chance. Fig 5 shows the enriched GO terms (p-value < 0.05) for each bin of target selectivity. We also show in the right column the total number of protein targets in PubChem BioAssay annotated with each term.

Several target selectivity bins are enriched with a characteristic set of MF GO terms. For example, FDA approved drugs active against oxidoreductase targets appear in a promiscuous bin (14–16.5), whereas drugs targeting binding proteins appear in a more selective bin (5–7.5). Overall, this result demonstrates that the different protein domains represented in PubChem BioAssay can be grouped into those druggable primarily with selective compounds, and those druggable primarily with promiscuous compounds. Interestingly, the patterns of term enrichment are different between the FDA approved and non-FDA compounds, with many of the top target classes druggable by more promiscuous FDA-approved compounds having primarily selective non-FDA active compounds and vice versa. This raises the question of if the selectivity levels characteristic of FDA approved drugs are a necessary property for those compounds therapeutic efficacy, or if more selective non-FDA compounds may also include viable drug candidates with a reduced chance of off-target effects. The highly enriched molecular function terms in some bins also raises the question of if these compound-target interactions may share a characteristic selectivity due to a shared chemical mechanism of bioactivity.

#### Stretched exponential distribution

The distribution of active targets for non-FDA compounds shown in Fig 4A show a very regular pattern, with a slight curvature in semi-log space. We found that this distribution is well described by the stretched exponential function shown in Eq 1 (*R*^2^ = 0.99912 for non-FDA cluster selectivity), where *c* and *x*_0_ are constant fit parameters, and the variable *x* represents the number of active protein targets. This is not due to the distribution of assay participation, as assay participation has a very irregular pattern with a large number of compounds screened against many targets as shown in Table 1 and S3 Fig in Supporting Information. Stretched exponential functions are commonly observed in natural multiplicative processes, and we report detailed methods and related citations for this observation in S1 Text of the Supporting Information [38].

$$P\left(x\right)=e^{-{\left(x/x_{0}\right)}^{c}}$$ (1)

#### Target selectivity and compound complexity

We investigated the distribution of target selectivities across compounds of different molecular sizes, and found that the overall distribution is similar. However, very large compounds tend to have fewer active targets, and FDA approved drugs are slightly smaller on average than non-FDA compounds (see S1 Text and S4 Fig). One possible explanation for this trend is that FDA approved drugs have been selected in the drug discovery process to be smaller than less optimized experimental drugs. The definition of molecular size used here is the quantity of non-hydrogen atoms. The largest FDA approved drugs tend to be natural products, which have several distinct patterns of target selectivity. For example, large antibiotics that evolved to inhibit prokaryotic ribosomal RNA structures tend to be extremely selective or inactive against protein targets, whereas many natural antimitotic and antiparasitic molecules are highly promiscuous. More detailed results of this molecular size target selectivity analysis are provided in S1 Text and S4 Fig of the Supporting Information.

### Promiscuous binders and hit ratio statistical model

#### Hit ratio model

Cross-reactive or “promiscuous” compounds are regarded as problematic in drug discovery efforts, as they show activity in a large fraction of HTS experiments, but fail to exhibit selective activity against the desired biological target(s) [8, 12, 27, 28]. Here we model the probability of a compound being promiscuous by estimating the hit ratio, *θ* with Bayes’ rule, based on its individual screening data. Hit ratio is the expected fraction of active targets that would be found if a compound were screened against the full target space represented in PubChem BioAssay. We model hit ratio with a binomial distribution, using a beta distribution conjugate prior in the manner developed by Dančík, V *et al.* (see “Hit Ratio Bayesian Model and Mixture Distribution” Methods section) [27]. This method enables filtering, and comparative ranking of compound promiscuity unbiased by individual assay participation.

By taking an equal number of random samples from the hit ratio posterior distributions for a set of compounds, we generate an equally weighted convex combination of hit ratio probabilities. This represents the probability of any individual compound from a set having a specific hit ratio, allowing us to compare the evidence for different hit ratios across different compound sets. Here we investigate the promiscuity of FDA approved drugs vs non-FDA compounds, and also investigate the ability of two common methods of identifying promiscuous compounds, pan-assay interference compound (PAINS) functional groups and promiscuous aggregator assays to distinguish between compounds that show selective vs promiscuous behavior in large bioactivity data [12, 28].

#### FDA approved vs non-FDA compounds

**Fig 6:**
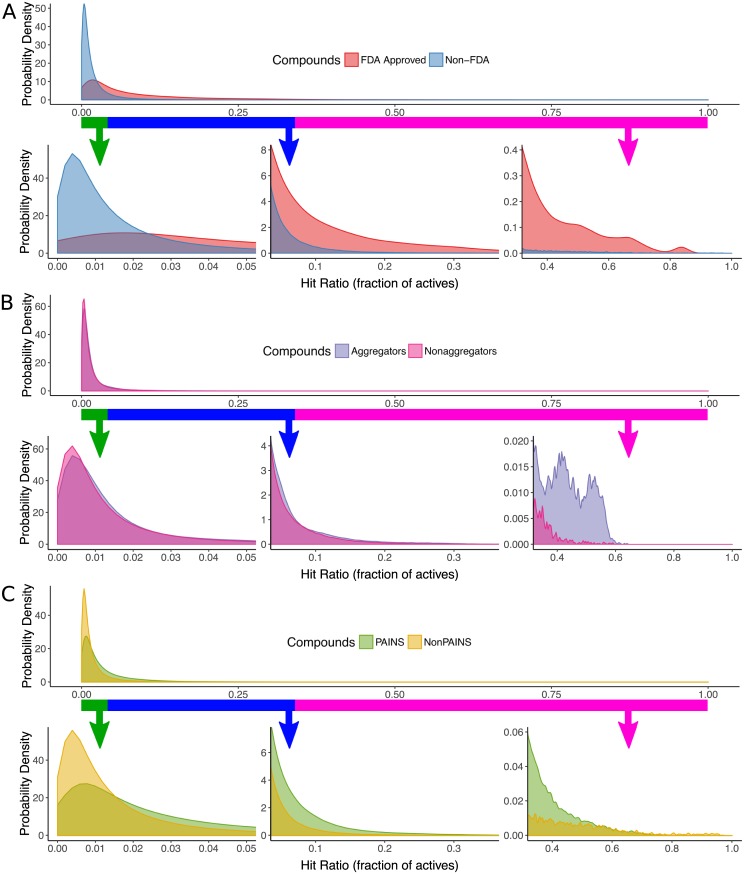
Mixture distribution of hit ratios. The probability density of hit ratios (*θ*) shown here, is an equally weighted convex combination of hit ratio probabilities for individual compounds, which represents the probability of any individual compound from a set having a specific hit ratio. Smoothing was applied to reduce sampling noise in low probability regions. The colored bars highlight a region of each probability distribution, with arrows pointing to a close-up plot of the probability density in that region. **(A)** Hit ratio distributions for FDA approved compounds vs non-FDA approved compounds. **(B)** Hit ratio distributions for aggregator compounds vs non-aggregators. **(C)** Hit ratio distributions for PAINS vs non-PAINS.

In Fig 6A we plot the hit ratio probability distributions for FDA approved and non-FDA compounds, computed as described above. The non-FDA compounds have a high probability density at low hit ratios (left side of plot), whereas the FDA approved drugs have much greater density at high hit ratios (middle and right of plot), consistent with the greater number of active targets described in the above “Target Selectivity Distribution” section. Quantitatively, for individual FDA approved drugs, there is an 85% probability of having a hit ratio below 17.8% (*P*(*θ*_*approved*_ < 0.178) = 0.85), while there is an 85% probability of a non-FDA drug having a hit ratio below a much lower threshold of 3.27% (*P*(*θ*_*other*_ < 0.0327) = 0.85). We also performed a two-sample Kolmogorov-Smirnov test, which measured a distance of *D* = 0.465 between the two probability distributions, indicating that the FDA approved and non-FDA compounds have nearly half of their probability density at different hit ratios. This test metric has a range between 0 and 1, indicating the maximum distance between the cumulative sums of the two probability distributions. Both the FDA approved drugs, and non-FDA compounds show a multimodal distribution dominated by highly selective compounds (left side of plot, approximately *θ* ≤ 0.05), a tail of middle range selective compounds (middle of plot, approximately 0.05 < *θ* ≤ 0.55), and a portion of promiscuous binders (right side of plot, approximately 0.55 < *θ*). The promiscuous binder tail among FDA approved compounds is dominated by drugs with well known promiscuous activity such as dasatinib (active against 145 out of 204 screened targets in the PubChem BioAssay data), sunitinib (active against 272 out of 313 screened targets), and morphine (active against 15 out of 16 screened targets) [39]. As cancers tend to exhibit robustness against inhibition of individual kinases, compounds which exhibit broad polypharmacology across the kinases are widely utilized in clinical oncology and represent many of the most promiscuous drugs [40].

#### Promiscuous aggregators

Promiscuous aggregators are small molecules that pose a significant challenge to high throughput screening, as they form colloidal aggregates that nonspecifically inhibit enzymes and other protein targets [12, 41]. To assess the ability of experimentally identified aggregators to distinguish between compounds with a high vs low hit ratio, we computed the hit ratio probability distributions separately for promiscuous aggregators and non-aggregators, as shown in Fig 6B. To facilitate this, we obtained a list of 1185 highly screened active aggregators and 55248 highly screened active nonaggregators previously identified by detergent-dependant inhibition of AmpC *β*-lactamase as reported by Feng *et al.* (see “Promiscuous Aggregators” in Methods) [41].

For aggregator compounds there is an 85% probability of having a hit ratio below 3.18% (*P*(*θ*_*approved*_ < 0.0318) = 0.85), while there is an 85% probability of a non-aggregator having a hit ratio below a slightly lower threshold of 2.73% (*P*(*θ*_*other*_ < 0.0273) = 0.85), showing that aggregators tend to be more promiscuous across the PubChem BioAssay data, but by a small margin. The maximum distance between the hit ratio probability distributions in cumulative probability space is *D* = 0.0596 as measured by a two-sample Kolmogorov-Smirnov test, demonstrating that a majority of aggregators and nonaggregators have a very similar overall hit ratio distribution compared to the distance of 0.465 between FDA approved and non-FDA approved drugs reported in the previous section. However, aggregators show high fidelity in identifying highly promiscuous compounds, as shown in the upper range of hit ratios in Fig 6B (right side of plot). This indicates that while most of the aggregators fail to show promiscuous activity across the PubChem BioAssay data, a large fraction of the most promiscuous compounds are identified as aggregators. We further investigate the ability of aggregators to identify promiscuous compounds in the “Sensitivity and Specificity of Aggregators and PAINS” section below.

#### Pan-assay Interference Compound (PAINS)

Pan-assay interference compounds (PAINS) are small molecules with substructural features that have been found to exhibit promiscuous activity across many high throughput screens, and may interfere with drug discovery efforts designed to identify target selective compounds [28]. We computed the hit ratio probability distribution separately for PAINS vs non-PAINS as shown in Fig 6C. We used the RDKit software library to identify 19988 PAINS compounds, and 298166 non-PAINS compounds, among the set of highly screened actives in PubChem BioAssay (see “Pan-Assay Interference Compounds” in Methods).

For individual PAINS compounds there is an 85% probability of having a hit ratio below 6.60% (*P*(*θ*_*approved*_ < 0.0660) = 0.85), while there is an 85% probability of a non-PAINS compound having a hit ratio below a lower threshold of 3.08% (*P*(*θ*_*other*_ < 0.0308) = 0.85), showing that PAINS tend to be more promiscuous than non-PAINS. The maximum distance between the hit ratio probability distributions in cumulative probability space is *D* = 0.228 as measured by a two-sample Kolmogorov-Smirnov test, demonstrating that PAINS have just under one quarter of their probability density at different hit ratios than non-PAINS. However, compared to the aggregators in Fig 6B, they show lower fidelity in identifying highly promiscuous compounds represented in the upper range of hit ratios (see right side of both plots). This suggests that promiscuous aggregators and PAINS may have mutually complementary utility for informing the curation of drug discovery libraries, as we investigate further in the next section.

By comparing the probability distributions in Fig 6A and 6C, PAINS have a hit-ratio distribution similar to, but somewhat less promiscuous than the FDA approved drugs. This raises a concern, as PAINS are most frequently used to eliminate non-viable drug candidates. However, we find that PAINS have a mean target selectivity count of 8.09 (median 4), but a median domain selectivity count only slightly lower, at 6.72 (median 4). Therefore, for PAINS compounds which are active against many targets, a substantially smaller fraction hit targets with common domains, as compared to the FDA approved drugs, as described in the above “Target Selectivity Distribution” section. This highlights a fundamental difference between PAINS and FDA approved drugs. While both tend to have activity against many targets, PAINS tend to be active against targets with unrelated sequences, while FDA approved drugs tend to be active against related targets.

#### Sensitivity and specificity of aggregators and PAINS

The highly screened active compounds can be divided into promiscuous and non-promiscuous categories based on the evidence in PubChem BioAssay, by choosing a promiscuity probability cutoff where *P*(*θ* ≥ 0.25) > cutoff. The number of promiscuous compounds at each cutoff is shown in the lower panel of S6 Fig in Supporting Information. For a given cutoff fraction, based on the public bioactivity data, our model predicts that approximately this fraction of compounds classified as promiscuous will have a true hit ratio above 0.25. We assessed the sensitivity (true positive rate) and specificity (true negative rate) of both PAINS and aggregators to categorize promiscuous compounds throughout a range of cutoffs from 0.01 to 0.9999. Here, sensitivity is defined as the fraction of compounds classified as promiscuous at a given cutoff that are also identified as PAINS or aggregators respectively, while specificity is the fraction classified as non-promiscuous that were also identified as non-PAINS or non-aggregators respectively.

PAINS showed a maximum sensitivity of 21% at a cutoff of 0.08, and aggregators showed a maximum sensitivity of 38% at a cutoff of 0.9996. Both non-PAINS and non-aggregators had a nearly constant specificity throughout this range, with non-PAINS having a specificity of 94%, and non-aggregators having a higher specificity of 98%.

As shown in the upper panel of S6 Fig in Supporting Information, the two have opposite trends where PAINS show decreasing sensitivity at increasing promiscuity cutoffs, while aggregators show increasing sensitivity at higher cutoffs. This is consistent with the probability distributions in Fig 6, in that both identify compounds with high hit ratios, but the PAINS compounds are more enriched in the middle range of hit ratios, while the aggregators tend to be highly promiscuous. While aggregators show both higher sensitivity and higher specificity, they identify a much smaller subset of promiscuous compounds that have extremely high hit ratios, and are not able to identify the large number of compounds with middle-range hit ratios that PAINS identifies.

Using a promiscuity probability cutoff of 0.5, our statistical model found 1409 promiscuous binders among the entire highly screened active PubChem BioAssay compound set, as shown in the center of S6 Fig in Supporting Information. Of these promiscuous binders, 1157 are not currently included among the set of PAINS or aggregators used here, and 75 are FDA approved drugs. The number of FDA approved drugs reduces to 24 with a higher promiscuity probability threshold of 0.999. As a resource for readers, we include the computed promiscuity probabilities *P*(*θ* ≥ 0.25) for all highly screened actives in Supporting Information S1 File, sorted by decreasing probability of promiscuity. This also serves to rank the compounds by target selectivity, with a ranking that is meaningful based on the experimental evidence, despite varying levels of assay participation.

### Comparison between annotated drug targets and public HTS data

We systematically compared the bioactivity data in PubChem BioAssay with the annotated targets of FDA approved drugs in DrugBank, in order to assess the level of agreement between the two, and identify the number of potential novel targets for the FDA approved drugs [25]. To enable this, we created a drug-target matrix encoding both bioactivity data and target annotations in a directly comparable manner. The rows represent the highly screened FDA approved drugs, while the columns represent all of the PubChem BioAssay screened and DrugBank annotated targets for these compounds. As many PubChem BioAssay activity results were generated with truncations of endogenous proteins, or using close orthologues to putative *H. sapiens* targets from other species, it was necessary to merge data from very similar targets, as described in the “Clustered Compound-Target Matrix” methods section. This resulted in 1829 distinct protein target columns, of which 1416 have a *H. sapiens* representative UniProt identifier. Each compound-target pair (position) in the matrix was assigned one of six possible values depending on its DrugBank annotation (annotated vs. unannotated) and its PubChem BioAssay activity results (untested, active, inactive). The resulting comparison between the BioAssay data and DrugBank annotations is shown in Table 6. There is a high level of agreement between the DrugBank target annotations and the PubChem BioAssay data, with 1082 compound-target pairs in agreement, and only 83 compound-target pairs in disagreement, where they are annotated as active in DrugBank but were found inactive in PubChem BioAssay. While the matrix is very sparse, with the majority of compound-target pairs both unscreened and unannotated, the PubChem BioAssay data substantially increases the density of the compound-target matrix, with 7817 active compound-target pairs not present in the DrugBank annotation, representing a new space of potential targets for these drugs. There are 867 protein target clusters (751 *H. sapiens*) that are annotated as active within DrugBank, however an additional 804 protein target clusters (576 *H. sapiens*) show activity in PubChem BioAssay but have no existing DrugBank annotation. Some of these active but currently unannotated targets may represent new target space that can be used to repurpose existing drugs for novel therapeutic purposes, or to explain currently unknown or unannotated targets in existing therapies. We provide a full list of these potentially novel drug-target pairs in S3 File of Supporting Information.

**Table 6:** Comparison of pubchem bioassay activity data to drugbank target annotations. All compound-target pairs for FDA Approved drugs are grouped into one of six possible categories. Depending on the HTS results in PubChem BioAssay, a compound-target pair is annotated as either untested, inactive, or active (rows in this table). Additionally, the compound-target pair is either annotated or unannotated as a known active target in DrugBank (columns in this table). Counts outside of parenthesis represent results against all protein targets, whereas counts to the right in parenthesis represent results against the subset in which the representative UniProt indentifer for each target cluster is from the *H. sapiens* proteome.

	Unannotated in DrugBank	Annotated Target in DrugBank
Untested in PubChem	1431855 (1111148 *H. sapiens*)	2097 (1900 *H. sapiens*)
Inactive in PubChem	153783 (115181 *H. sapiens*)	83 (83 *H. sapiens*)
Active in PubChem	7817 (6848 *H. sapiens*)	1082 (1008 *H. sapiens*)

While the false positive rate of these PubChem BioAssay activity outcomes is not precisely known, our estimate above using replicated assay pairs suggests that the number of false positives is less than, and of the same order of magnitude as the number of true positives. Consequently, we predict that at least half of these novel drug-target activity results are experimentally repeatable. Additionally, as we demonstrate in the next section, many of these new currently unannotated active values fall into dense biclusters, where the same compound has been found active against a large number of closely related protein targets across many assays. As these biclusters are highly enriched for a large number of active scores, these are unlikely to be a result of random error. We provide a full list of these high confidence biclusters in S4 and S5 Files of Supporting Information.

### Drug-Target (DT) biclustering analysis

#### Biclustering overview

To investigate the possibility of shared patterns of activity between sets of FDA approved drugs and their protein targets, we created a drug-target binary activity matrix based on the drug-target matrix in the above section. Each drug-target combination was assigned a value of 1 if active in PubChem BioAssay, or annotated as a known target in DrugBank. Untested or inactive values are assigned a value of 0. The resulting bioactivity matrix has a total active in PubChem BioAssay and/or annotated as active in DrugBank score density of 0.69%. We then clustered this matrix using the BicBin sparse biclustering algorithm (see methods) [42]. This type of clustering algorithm clusters rows and columns simultaneously allowing us to identify both sets of compounds and targets sharing similar activity profiles within each dimension. BicBin was chosen among several biclustering algorithms as it finds sparse biclusters with flexible options, scales to large matricies, and finds top-scoring clusters first. We identified the 16 highest scoring biclusters which contained at least two compounds and at least two targets as shown in Table 7. These biclusters had an activity density substantially higher than the entire matrix, ranging from 31.46% to 92.19%. These biclusters contain 406 drugs, of which 136 appear in multiple biclusters with a maximum of 6 biclusters per compound, and 346 unique representative protein targets, of which 107 appear in multiple biclusters with a maximum of 4 biclusters per protein. S1 Fig in Supporting Information shows the entire matrix represented as a bipartite graph with compounds colored by their highest scoring bicluster (white if unclustered), and protein targets in black. We found that the very sparsely connected graph clusters into very densely connected biclusters where a sizable set of distinct drugs has been found to be active against a sizable shared set of distinct targets. To functionally annotate each bicluster, we identified the most common Pfam domains present in their protein targets [32]. As shown in Table 7, in some biclusters most or all of the protein targets share a common domain that is the likely the target of these compounds, *e.g.* 22 out of 27 targets in bicluster 1 share the rhodopsin-like receptor domain (PF00001: 7 transmembrane receptor). Fig 7 visualizes the compound-target activities in bicluster 1 as a heatmap. In other biclusters, only a small fraction share a common domain such as bicluster 15 where only 6 out of 57 targets share PF00001. These cases warrant deeper investigation as to why they share a common activity pattern, but with a more heterogeneous domain composition. This questions is investigated in the next section. As mentioned in the previous section, we provide a full list of these biclusters in S4 and S5 Files of Supporting Information.

**Table 7:** Top pfam domains in each bicluster. Shown are the top 16 highest scoring drug-target biclusters with more than one compound and more than one target. The number of drugs (cids) and targets is shown in columns 2 and 3, respectively. The 4th and 5th columns give the name of the most abundant domain and its frequency, respectively. The last (6th) column shows the BicBin score, representing the density and size of the bicluster. The BicBin score is the negative exponent of the Chernoff Bound. It is inversely proportional to the probability of each bicluster occurring by random chance, as described in Methods.

#	Compounds	Targets	Top Pfam Domain	W/ Domain	Score
1	62	27	PF00001 7 transmembrane receptor (rhodopsin family)	22	13.35
2	130	5	PF00067 Cytochrome P450	5	11.61
3	119	9	PF00104 Ligand-binding domain of nuclear hormone receptor	6	9.52
4	53	18	PF00194 Eukaryotic-type carbonic anhydrase	9	7.28
5	4	97	PF00069 Protein kinase domain	89	7.28
6	10	24	PF00001 7 transmembrane receptor (rhodopsin family)	13	5.97
7	48	23	PF00001 7 transmembrane receptor (rhodopsin family)	15	5.61
8	63	28	PF00001 7 transmembrane receptor (rhodopsin family)	7	4.79
9	2	67	PF00069 Protein kinase domain	49	4.46
10	2	78	PF00001 7 transmembrane receptor (rhodopsin family)	10	4.05
11	8	8	PF00484 Carbonic anhydrase	6	4.76
12	6	13	PF00520 Ion transport protein	4	3.91
13	57	4	PF00001 7 transmembrane receptor (rhodopsin family)	2	4.36
14	18	5	PF00001 7 transmembrane receptor (rhodopsin family)	5	4.78
15	2	57	PF00001 7 transmembrane receptor (rhodopsin family)	6	3.89
16	12	16	PF00817 impB/mucB/samB family	3	3.84

**Fig 7:**
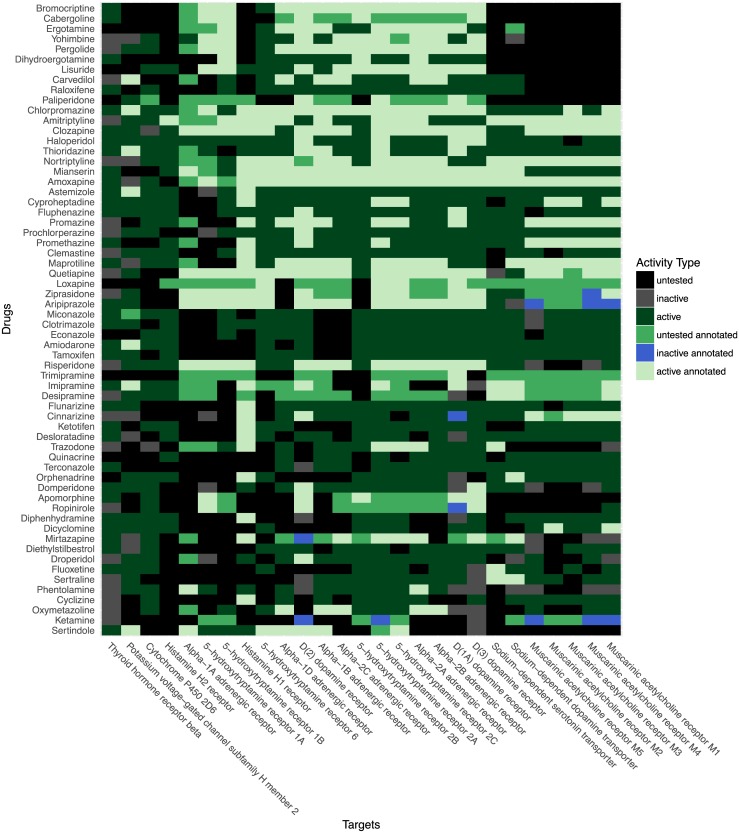
Bioactivity of drug-target bicluster 1. The vertical axis lists the drugs in this bicluster by common name, and the horizontal axis represents the UniProt names for the representative targets of each sequence-similar target cluster. The compound-target pairs are colored according to one of six colors: untested in PubChem BioAssay (black), inactive in PubChem BioAssay (grey), active in PubChem BioAssay (dark green), untested but annotated as active in DrugBank (green), inactive in PubChem BioAssay but annotated as active in DrugBank (blue), and active and also annotated as active in DrugBank (light green). Rows and columns are sorted by bioactivity profile similarity.

#### Drug-Target (DT) bicluster GO slim analysis

**Fig 8:**
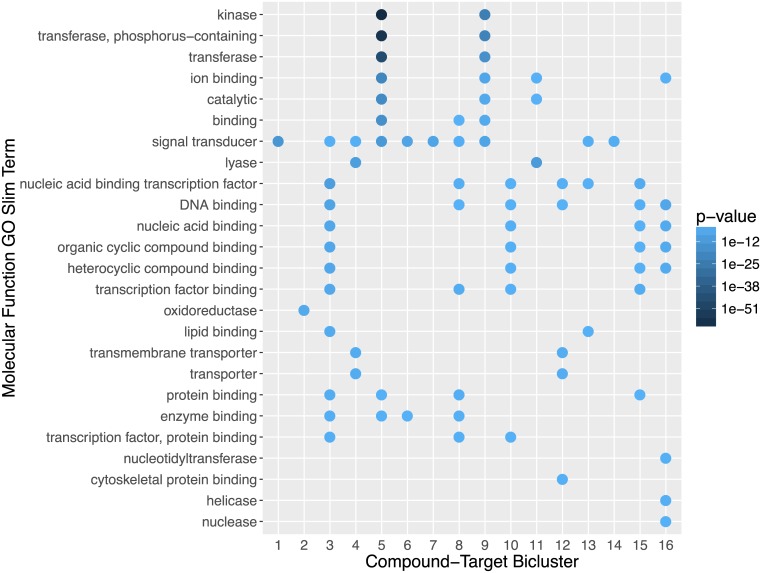
Molecular Function Gene Ontology Slim (MF GO Slim) term enrichment for each drug-target bicluster. Enrichment measured by hypergeometric test. Terms with *p* ≤ 0.05 are shown and sorted increasingly.

To further categorize each bicluster by functional processes, we performed an enrichment analysis of the Molecular Function GO Slim terms associated with the representative protein targets within each bicluster. Fig 8 shows the most enriched terms for each bicluster. Most biclusters exhibit a distinct pattern of enriched GO terms, distinguishing them from other biclusters. For example, bicluster 5 consists of four kinase inhibitor drugs with known broad kinase-activity (Dasatinib, Sorafenib, Erlotinib, and Gefitinib), and a highly enriched kinase GO term (PF00069, p-value 9.11 * 10^−64^) present in the annotation of 89 out of a total of 97 targets in this bicluster. Additionally if the entire drug-target network is colored by the GO terms present in each target, a distinct regional pattern emerges, where targets sharing active compounds also tend to share common GO terms as shown in S2 Fig of Supporting Information. Overall the GO Slim annotations provide a more informative functional summary of each bicluster than the Pfam annotations. This is often the case because a greater portion of the targets in each bicluster tends to share the most enriched GO term, but not necessarily a specific Pfam domain.

As the top scoring biclusters listed here include approximately half of the highly screened active FDA approved compounds, they constitute specific examples which partially explain the higher number of active targets among FDA approved drugs compared to non-FDA compounds (consider Fig 4, as well as the greater probability density at higher hit ratios in Fig 6A). In summary, a substantial fraction of the FDA approved compounds show broad activity across a large set of related targets in the same bicluster, which are enriched for common Pfam domains and/or MF GO slim terms.

#### Compound structure vs bioactivity bicluster analysis

**Fig 9:**
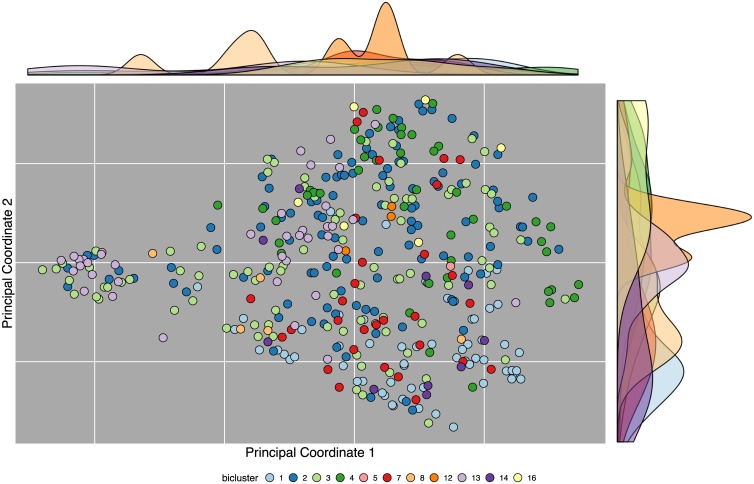
Bicluster (color) vs compound structure (position). Multidimensional scaling (MDS) was used to embed small molecules into a two dimensional space (x- and y-axis). Each point represents an FDA approved drug. A density map colored by each bicluster is shown for the MDS principal coordinate 1 (on top) and principal coordinate 2 (on right). The distance between the points is proportional to the chemical similarity between the two compounds. Bioactivity-based biclustering results are also indicated by colors, with each compound assigned to its lowest numbered (densest and/or largest) bicluster. Only the 11 biclusters with the largest number of compounds are shown, to allow for a visually distinct color palette.

In order to compare the compound structure vs bioactivity patterns among these biclusters, we clustered the FDA approved drugs by structural similarity using atom pair (AP) descriptors and the Tanimoto coefficient as similarity metric [43]. Fig 9 shows the compounds from the 11 largest bioactivity biclusters, positioned according to structural similarity, and colored according to their lowest numbered (densest and/or largest) bioactivity bicluster. The structural distances were used to project each compound into two dimensional plane with multi-dimensional scaling (MDS) where the points (compounds) are spaced proportionally to the chemical structure difference between the compounds, with more similar compounds closer together. Visually, two distinct patterns can be identified where structurally similar compounds (in close proximity) also cluster together with similar bioactivity (*e.g.* bicluster 1 shown in light blue, a cluster of primarily aromatic compounds active against G-protein-coupled receptor targets). However, the opposite can also be observed, where compounds with very similar bioactivity have diverse structures (*e.g.* bicluster 3 shown in light green, a cluster with many nuclear receptor targets).

To estimate the extent to which the structure-function principle (*i.e.* that similar structures have similar bioactivities) applies to this data, we clustered the 406 compounds represented in the biclusters into discrete clusters using complete linkage hierarchical clustering with subsequent tree cutting with *k* = 11. The latter value matches the number of biclusters remaining when the compounds are each assigned to a unique bicluster. To quantify the similarity among the structural clusters and the bioactivity clusters from of the above biclustering section, we compared the numbers of identical and unique compound pairs appearing in the two clustering results using the Jaccard index. The result indicated that 15.21% of compound pairs were joined into clusters by both methods. If the structural clustering is replaced with a random grouping into one of 11 clusters weighted by the cluster size distribution in the structure clustering, we see a mean Jaccard index of only 11.10% (*sd* = 0.39% and permutation p-value 0.0001) across 10,000 random clusterings. This quantifies what can be seen visually in Fig 9, that overall structural similarity correlates with bioactivity similarity, but with a sizable number of exceptions.

### Target-Protein (TP) network

In order to extend the drug-target biclustering analysis shown above to the full set of PubChem BioAssay bioactivity data, we created a Target-Protein (TP) network where proteins are connected if they are targeted by over 50% of the same non-promiscuous compounds (as described in Methods). This was inspired by the TP network previously published by Yildirim *et al.*, while adding a bioactivity similarity threshold and excluding promiscuous compounds, in order to enable the incorporation of large primary screening data while limiting spurious edges [44]. This graph approximates the structure of a full compound-target binary activity matrix (or bigraph), in a computationally efficient manner by excluding the small molecule nodes.

**Fig 10:**
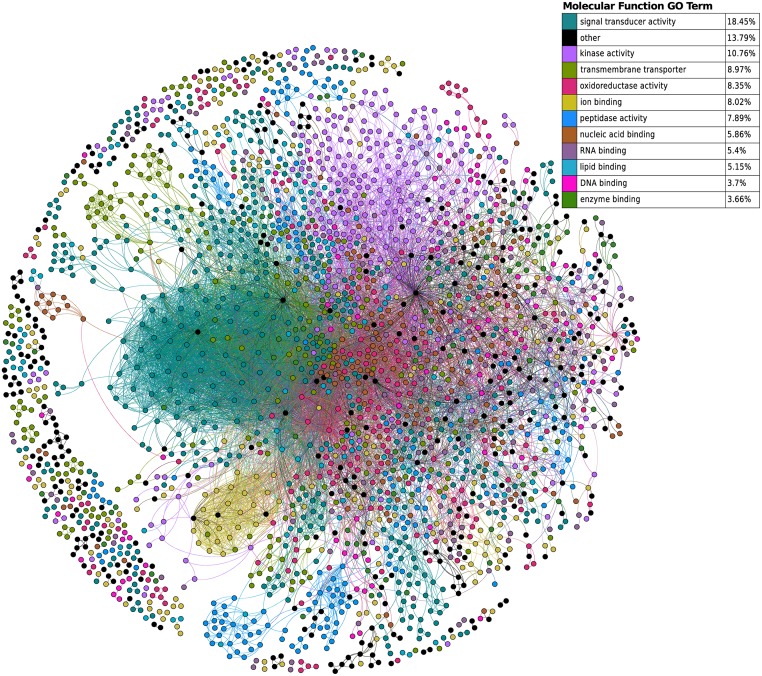
Target-protein network and Molecular Function Gene Ontology Slim (MF GO Slim). Each node represents a protein target, and edges connect any two protein targets with greater than 50% bioactivity similarity across non-promiscuous binding compounds. Targets are colored according to MF GO Slim terms, with unannotated targets colored black. Shown are 2407 nodes (target proteins with at least one edge) and 11317 edges representing shared bioactivity among the mutually screened subset of the 1.2M compounds tested in the bioassays we analyze in this study.

Fig 10 contains a visualization of the entire Target-Protein (TP) network. It has 2407 nodes (target proteins with at least one edge) and 11317 edges. There are 176 connected components with the majority of nodes in the largest. The average degree is 9.40 with a graph density of 0.004. In Fig 10, protein targets are colored according to the 11 most abundant Molecular Function Gene Ontology Slim (MF GO Slim) terms among the PubChem BioAssay protein targets, with nodes lacking any of these 11 terms labeled in black (other). If a target is annotated with more than one of these 11 terms, the most specific term was chosen as the representative color for that node. Fig 10 demonstrates a distinct grouping of protein targets with shared bioactivity by MF GO terms. As the structure of the network was not informed with MF GO annotations, this indicates that the overall pattern demonstrated above for the drug-target biclustering analysis also extends to the full set of compounds and targets in the PubChem BioAssay data, in that targets sharing a common MF GO slim annotation tend to have a distinct but shared set of active small molecules.

## Methods

Most analysis steps were performed with the open source software R, bioassayR and ChemmineR. The latter two are Bioconductor packages developed by the authors. bioassayR was co-developed alongside this study to support large scale bioactivity data analysis, and is provided as a resource to readers at http://bioconductor.org/packages/bioassayR/. Several of the bioactivity methods are described in the bioassayR paper by Backman *et al* [35]. The full source code of the analysis presented in this paper is freely available online at http://github.com/girke-lab/targetSelectivity on GitHub and http://doi.org/10.5281/zenodo.220994 on Zenodo.

### Bioactivity database

We used the R package bioassayR to build a database which contains all small molecule bioactivity screens from PubChem BioAssay which include at least one real activity score (active or inactive) and have a single protein target specified. Both raw numeric scores, and discrete active/inactive categories were parsed, and stored in the database, however direct cross-comparison between the numeric scores is limited by varying assay designs and scoring methods.

### Clustering protein targets by sequence

We used the kClust tool to cluster the non-redundant set of both the protein targets in PubChem BioAssay as well as the protein targets interacting according to DrugBank with FDA approved drugs [25, 34]. Stringent threshold settings were chosen to merge very close orthologues, paralogs, and engineered proteins (*e.g.* truncations performed for screening purposes). The parameters used were *s* = 2.93, E-value ≤10^−4^, *c* = 0.8. For each resulting cluster, a single representative protein was chosen for annotation purposes. These representatives were chosen with the following order of precedence: an annotated *H. sapiens* drug target (from DrugBank), any *H. sapiens* target with a known UniProt identifier, a non *H. sapiens* target with a known UniProt identifer, and lastly a non *H. sapiens* target with only a GenBank GI number and no known UniProt identifier.

### Clustering the compound-target matrix

We used bioassayR to generate a compound-target binary sparse matrix summarizing a substantial fraction of the protein target bioactivity data in PubChem BioAssay. Only compounds screened against at least 10 protein targets (distinct GenBank GIs) were included, in order to avoid biasing the selectivity analysis by compounds with too limited data. In order to reduce the sparseness and duplication in these data, assays sharing identical protein targets, or targets falling into the same sequence cluster (see above) were merged into a common column. The merging was performed in a way where active scores take precedence over inactive scores. Each column was annotated by a representative protein for that target cluster, as described above.

### Protein annotations and GO enrichment

Pfam-A (version 29.0) domains were mapped to target proteins with HMMER3 (version 3.1b2) [32, 45]. An E-value ≤0.01 was used as domain reporting threshold. The target proteins included all PubChem BioAssay targets, DrugBank annotated targets, and the *H. sapiens* reference proteome (proteome ID UP000005640) provided by UniProt [46]. Gene Ontology annotations for protein targets were obtained from UniProt, while Gene Ontology annotations for Pfam domains were obtained from InterPro [47]. The subset of GO slim terms (Generic version) was obtained from the GO Consortium. Hypergeometric GO term enrichment tests were performed using the R language GOstats and GSEABase packages [48].

### BicBin biclustering of drug-target matrix

The BicBin algorithm was used to identify bioclusters iteratively using the parameters *α* = 0.6, *β* = 0.6, representing no bias between adding compounds or targets [42]. These thresholds were chosen to find the largest possible biclusters, without merging biclusters that share little or no overlapping activity. The BicBin biclustering algorithm used here finds dense biclusters of compound-target activity by scoring them with the multiplicative version of the Chernoff Bound applied to the Binomial distribution, which estimates the upper limit of the probability of these clusters occurring by random chance [42]. The bicluster scores shown in Table 7 represent the negative exponent of the Chernoff bound, and therefore higher scores correspond to lower probabilities, and therefore larger and denser biclusters. Biclusters were found and scored iteratively, by first zeroing out the previous biclusters.

### Hit ratio bayesian model and mixture distribution

We model hit ratio *θ* of each compound with a binomial distribution, using a beta distribution conjugate prior in the manner developed by Dančík, V *et al* [27]. For a given number of active targets *n*, out of *N* screened targets, we assume that *n* has a binomial distribution, as in Eq 3. We then apply Bayes’ rule (Eq 2) to compute the posterior *P*(*θ*|*n*) with a beta distribution conjugate prior as in Eq 4. The values of *α* and *β* are computed from the mean *μ* and standard deviation *σ* of hit ratios among all active compounds screened against at least 20 distinct targets, using the bioassayR function *crossReactivityPrior* and Eqs 5 and 6.

$$P\left(\theta |n\right)=\frac{P(\theta )P(n|\theta )}{P(n)}$$ (2)

P(n|θ)=Nnθn(1-θ)N-n (3)

$$P\left(\theta |\alpha ,\beta \right)=\frac{\Gamma (\alpha +\beta )}{\Gamma (\alpha )\Gamma (\beta )}\;\theta^{\alpha -1}{\left(1-\theta \right)}^{\beta -1}$$ (4)

$$\alpha =\mu^{2}\left(\frac{1-\mu }{\sigma^{2}}-\frac{1}{\mu }\right)$$ (5)

$$\beta =\alpha \left(\frac{1}{\mu }-1\right)$$ (6)

We then compute the probability of a compound being a promiscuous binder *P*(*θ* ≥ 0.25) with the bioassayR function *crossReactivityProbability*, and can obtain random samples from the posterior distribution of each compound with the R language function *rbeta*. To obtain an equally weighted convex combination of hit ratios for a compound set, we took an equal number of samples with *rbeta* for each compound, and then took one million unbiased samples without replacement from these. To plot this distribution we used the *geom_density* function of the ggplot2 software library with the option *adjust* = 3 to smooth sampling noise in the tails.

The Bayesian hit ratio model is based on an underlying assumption that the available activity data for a given compound represents activity against a random sample with replacement of the screenable protein target space. While this is a reasonable approximation for compounds screened against a large number of diverse targets, in many cases compounds screened against a small number of targets are likely to have substantial bias in their target set. Therefore, there is a strong possibility that a compound with only one or two active targets is highly selective, or is an inactive compound with a false positive, resulting in overfitting that would make the computed hit ratio an overestimate. As reported by Jasial *et al.*, these compounds with a small number of active targets are unlikely to exhibit undiscovered promiscuity or activity against many additional targets as they are screened in an increasing number of assays [26]. For this reason, here we model hit ratio primarily to identify compounds with a large number of active targets (polypharmacological and promiscuous compounds), while looking at the absolute number of active targets when investigating highly selective compounds, as shown in the “Target Selectivity Distribution” section. Additionally, by using only highly screened compounds, we avoid both many cases of overfitting, and avoid plotting probability distributions for compounds with highly uninformative data, that would result in a non-localized probability density function.

### Promiscuous aggregators

We obtained a list of known promiscuous aggregator and nonaggregator small molecule PubChem compound identifiers (cids) by referencing PubChem BioAssay assays #584 and #585 as described by Feng *et al.* [41] These assays together identify detergent-dependant inhibitors of AmpC *β*-lactamase. We obtained the list of promiscuous aggregators by identifying compounds marked as active in the assay without detergent (#585), but inactive in the assay with detergent (#584). The list of nonaggregators includes both inhibitors active in both assays, and noninihibitors inactive in the assay without detergent. We excluded from consideration all compounds which obtained an inconclusive result in either assay, or were not highly screened, having been tested in PubChem BioAssay against less than 10 distinct targets. We also excluded compounds without activity against at least one protein target in PubChem BioAssay. This resulted in a list of 1185 promiscuous aggregators, and 55248 nonaggregators.

### Pan-Assay Interference Compounds (PAINS)

We used the RDKit software library (version 2016.03.1) SMARTS based PAINS filters to identify compounds classified by the PAINS filters A, B, or C. These SMARTS filters are based on the SMARTS conversion published by Saubern *et al.* based on the SLN format filters originally published by Baell *et al.* [28, 49] This identified 19988 PAINS compounds, and 298166 non-PAINS compounds, among the set of highly screened actives in PubChem BioAssay. 68 of the compounds we identified as PAINS are also FDA approved drugs. An additional 7814 compounds had structures we could not parse with RDKit and were excluded.

### Target-Protein (TP) network and network visualizations

Targets were connected by bioactivity profile similarity using the *trinarySimilarity* function of bioassayR, with default options. This computes Tanimoto similarity coefficients between bioactivity profiles, by considering only commonly tested compounds. The Tanimoto, as computed here, is the size of the intersection divided by the size of the union of active compounds between the two targets. If the pair of targets did not share at least 12 mutually screened compounds, or at least 3 actives, we categorized this pair as having insufficient evidence, and assigned a similarity value of 0. The similarity matrix was converted to a binary connection matrix based on a similarity value of at least 0.50, and then converted to a graph object with the R package igraph [50]. All network visualizations were generated with Gephi using the ForceAtlas2 layout algorithm [51, 52]. Because the layout engine itself was not provided with any annotation information (color), the color based groupings are solely based on the level of connectivity.

We did not exclude infrequently screened compounds as in the other sections in this study, as this analysis was able to make meaningful use of those compounds. We found the overall structure of the graph is roughly the same at different similarity thresholds, however we chose this higher cutoff to reduce the number of edges in the visualization. This high evidence threshold also avoids spurious edges resulting from false positive activity outcomes. Despite such a high cutoff, the majority of the graph is highly connected, showing that a large number of target pairs share very similar activity profiles across a large number of small molecules. Compounds were excluded if their probability of promiscuous binding was greater than 50% (*P*(*θ* ≥ 0.25) > 0.5). This resulted in the exclusion of 29179 compounds. As this analysis was not limited to highly screened compounds, this is a much higher number than the quantity of highly screened promiscuous compounds reported above. Out of all protein targets in PubChem BioAssay, only 2249 had at least one edge. Removing this small fraction of promiscuous binding compounds (2.52% of total compounds) substantially reduced the number of edges in the network. The number of node pairs (edges) with a computable similarity (enough shared actives or mutually screened targets) above 50% dropped from 283353 to 194444 and the number of highly similar node pairs we connected with edges dropped from 84298 to 9854.

### De-duplication of single domain clusters

As outlined above, clustering of target proteins by the presence of single Pfam domains results frequently in duplicates of nearly identical clusters for multi-domain proteins. To eliminate this redundancy in most figures and tables, we also included domain composition clustering. For this we generated a list all single and multiple domain mappings in the target proteins of PubChem BioAssay using the same mapping parameters as described above under “Protein Annotations and GO Enrichment.” We provide this list as a downloadable resource to readers in S8 File of Supporting Information. Next, for the set of domains presented in a given table or figure, we created an undirected graph, where each domain is a node, and each edge represents a pair of domains which were found together in at least one target protein. Then we iteratively identified the largest clique (fully connected subgraph), while removing all nodes from this clique for the next iteration. These cliques identify the largest independent sets of domains which have all been found to co-occur. Finally, we chose a single domain to represent this cluster in the corresponding figure or table. For each clique, we chose the domain which occurs in the greatest number of screened targets. Note that this de-duplication was performed only within plots and tables, and that the results without de-duplication are provided for download in S2 File of Supporting Information.

## Conclusion

By systematically analyzing a large volume of public bioactivity data, we highlight several new patterns of bioactivity that may prove useful for informing drug discovery efforts. We also provide additional context to the previously reported finding that FDA approved drugs are, on average, active against a greater number of targets than non-FDA approved active molecules identified by HTS methods [24, 26]. We show that this greater number of targets is not due to biased assay participation, both by using a statistical model which considers the evidence for each compound individually, and by looking at the mean and median assay participation. While still noticeable, the difference in the number of active targets between FDA approved and non-FDA compounds decreases substantially if proteins sharing very similar amino acid sequences, or common domains are not counted separately.

As demonstrated by the high number of previously unannotated active targets (Table 6), and the high density of drug-target activity biclusters (Table 7 and Fig 8), several sets of FDA approved drugs exhibit activity across a shared set of related targets. Previous literature suggests several plausible explanations for how these drugs may have similar or identical bioactivity profiles, while inducing distinct therapeutic phenotypes *in-vivo*. For example, it has been demonstrated that several common drug target receptor families exhibit biased signaling, where a given receptor can activate a large number of downstream processes, in different ratios unique to a given ligand structure, tissue, and organism state [53, 54]. Additionally, bioavailability and biological compartmentalization can limit the *in vivo* access of a small molecule drug to only a small fraction of the targets it may show activity against *in vitro* [55]. Complex network effects and biological feedback can also cause a drug interacting with multiple targets to exhibit functional selectivity. For example, Lehar *et al.* (2009) published an analysis of synergistic drug combinations, showing that combinations of multiple drugs acting against different targets in the same pathways tend to induce a phenotype at lower doses, with lower incidence of off-target effects [56]. Lastly, binary active/inactive HTS data may fail to resolve different receptor binding kinetics that would cause a drug to exhibit target selectivity in the context of a specific dosage level. For example, drugs are often classified and evaluated according to therapeutic index (TI), or the ratio between the dose that results in toxicity to the dose that produces a desired efficacy [57]. For low TI drugs where the desired effect and toxicity are mediated with different receptors that have only slightly different binding affinities, binary active/inactive data could be expected to report activity for both the therapeutic and toxic targets.

This cross-reactivity we observe in FDA approved drugs raises the question and possibility of exploiting this pattern to identify viable drug candidates in noisy and error prone HTS data. With false positives occurring at the same order of magnitude as true positive bioactivity outcomes, it is likely that a substantial fraction of singular active values are due to experimental error. For some drug discovery efforts against target classes where the FDA approved drugs tend to show cross-reactivity within a protein family, it may be appropriate to regard targets sharing a common Pfam domain or molecular function annotation as replicates, and libraries can be enriched for broad activity within this category, while removing both highly-selective compounds, and promiscuous binders active against a large fraction of the screened targets.

We demonstrate that these data contain a large number of novel active targets for FDA approved drugs, a large number of novel compounds active against known drug targets, and a large set of novel compound-target pairs with no evidence of druggability by FDA approved drugs. By quantifying the rate of agreement between replicated pairs of compound-target activity outcomes, we estimate that less than half of these novel outcomes are due to experimental and data curation errors, and therefore may represent a valuable resource for further drug discovery efforts.

Additionally, we use the statistical model mentioned above to score all highly screened active compounds in PubChem BioAssay by their probability of being promiscuous binders given the available data, and assess the ability of PAINS and aggregators to identify the most experimentally promiscuous compounds. We find that both methods offer mutually complementary strengths at identifying different sets of promiscuous binders, and we also report 1157 compounds with a greater than 50% chance of being promiscuous, that were not included among the sets of known PAINS or aggregators we used for our analysis. We provide the promiscuity probability values for all highly screened active compounds, as well as the source code and results for these analyses as a reference to readers, with the hope that they will contribute to the discovery of medically and biologically useful small molecules.

## Supporting information

S1 Text Fully screened sub-matrix, error rate, selectivity by molecular size, and stretched exponentials. This text contains more analysis details on the fully screened sub-matrix we provide as a downloadable reference, an algebraic estimate of error rates, our target selectivity by molecular size analysis, and a more in-depth discussion and methods for the stretched exponential selectivity distribution.
(PDF)

S1 Fig Drug-Target (DT) bipartite network biclusters. Protein targets are shown in black, with FDA approved drugs shown in color, based on their bioactivity bicluster. Unclustered compounds are shown in grey. No color key is provided, as some colors were reused in order to visualize a large number of biclusters. Node position is based on connectivity, with the same positions as in S2 Fig.
(PDF)

S2 Fig Drug-Target (DT) bipartite network Gene Ontology (GO). FDA approved drugs are shown in black, with protein targets show in color based on the most specific Molecular Function GO Slim term for each target. Unannotated targets are shown in white. No color key is provided, as some colors were reused in order to visualize a large number of GO terms. Node position is based on connectivity, with the same positions as in S1 Fig.
(PDF)

S3 Fig Distribution of distinct protein target assay participation. Data is included from all assay experiments in PubChem BioAssay annotated with one or more clearly defined protein targets, and reporting an active score for at least one small molecule. The dashed vertical line is drawn at 10 targets, which is the minimum value we categorize in this study as a “highly screened” compound.
(PDF)

S4 Fig Target selectivity by molecular size. Violin plot with horizontal lines drawn at the 0.25, 0.5, 0.75 quantiles with tails trimmed to the range of data, as described in the “Target Selectivity by Molecular Size” section of S1 Text. Molecule size is quantified here by the number of non-hydrogen (heavy) atoms. (A) Target selectivity vs. molecular size across the entire range (y axis) of variation in these data. (B) Target selectivity vs. molecular size zoomed in on the y-axis to show more detail.
(PDF)

S5 Fig Selectivity distribution. The distribution of cluster selectivity counts for non-FDA approved compounds as shown in Fig 4, along with best fit lines using two-parameter versions of the exponential, power law, and stretched exponential functions, as described in the “Stretched Exponential Selectivity Distribution” section of S1 Text. The stretched exponential fits the data better than the exponential, or power law functions (with *R*^2^ = 0.99912, 0.99131, and 0.97916 respectively).
(PDF)

S6 Fig Sensitivity of PAINS and aggregators vs promiscuity probability cutoff. The top panel shows the sensitivity (true positive rate) of PAINS and aggregators to categorize promiscuous compounds throughout a range of promiscuity probability cutoffs *P*(*θ* ≥ 0.25) > *x* over the range *x* = [0.01, 0.9999]. The bottom panel shows the number of promiscuous compounds at each cutoff value. While the Bayesian model classifies all highly screened compounds, the values shown in the top panel are computed only with the subsets that were classified as aggregators/non-aggregators, and PAINS/non-PAINS respectively. There is an intersection of 44 compounds in this analysis which are classified as both PAINS and aggregators, out of 56330 highly screened active compounds which were tested and had valid results in both.
(PDF)

S7 Fig Target selectivity distribution among targets sharing a common protein domain. The distribution of active and tested targets for FDA approved and non-FDA approved compounds within targets sharing a common Pfam domain, as described in the “Target Selectivity Distribution Among Targets Sharing a Common Protein Domain” section of S1 Text. See Table V in S1 Text for the full names of each domain, as well as the number of FDA approved compounds, non-FDA compounds, and total protein targets for each domain. Horizontal lines are positioned at the 25%, 50%, and 75% quantiles for each distribution, with whiskers extending to 1.5 times the inter-quartile range. **(A)** The distribution of active protein targets within each domain. **(B)** The distribution of total screened targets within each domain. **(C)** The same as A except with iterative random removal of activity outcomes from the most highly screened FDA Approved drugs, such that the median number of screened targets for the FDA approved compounds is equal to or slightly less than that for non-FDA approved compounds, to enable cross-comparison without bias due to screening volume. **(D)** The same as B except with iterative random removal of activity outcomes from the most highly screened FDA Approved drugs, such that the median number of screened targets for the FDA approved compounds is equal to or slightly less than that for non-FDA approved compounds, to enable cross-comparison without bias due to screening volume.
(PDF)

S1 File Target selectivity, cluster selectivity, domain selectivity, and promiscuity probability *P*(*θ* ≥ 0.25) for all highly screened active compounds. This is a zipped Excel readable tab separated text file with PubChem compound ids (cid) for each compound in the first column. Compounds are sorted in order from most promiscuous, to most selective. This also serves as a ranked list of target selectivity in reverse order.
(ZIP)

S2 File List of pfam domains including median target, cluster, and domain selectivities for FDA approved and non-FDA compounds. This is a zipped Excel readable tab separated text file with Pfam identifiers for each domain in the first column. This is the full data shown in Tables 4 and 5, including non-*H. sapiens* domains. All domains with at least one active compound are included. We also include the number of active FDA approved and non-FDA approved compounds as shown in Fig 3.
(ZIP)

S3 File Potentially novel targets for FDA-approved drugs. This is a zipped Excel readable tab separated text file with PubChem compound ids (cids) for each compound in the first column, and a representative UniProt protein target identifier for each sequence-similar target cluster in the second column. These represent compound-target pairs reported as active in PubChem BioAssay, but not represented among the DrugBank annotated targets list. Several targets had no UniProt translation and include a GenBank GI number instead, prefixed with “gi_”.
(ZIP)

S4 File FDA approved drug biclusters. This is a zipped Excel readable tab separated text file with PubChem compound ids (cids) for each compound in the first column, and a bicluster for each compound in the second column corresponding to the drug-target biclusters described in the text.
(ZIP)

S5 File Protein target biclusters. This is a zipped Excel readable tab separated text file with a representative UniProt protein identifier for each sequence-similar target cluster in the first column, and a bicluster for each in the second column corresponding to the drug-target biclusters described in the text. Several targets had no UniProt translation and include a GenBank GI number instead, prefixed with “gi_”.
(ZIP)

S6 File Target-protein network. This is a Gephi readable zipped GML (Graph Modeling Language) formatted file, which contains the target-protein network described in the manuscript. Each node (protein) is labeled with a GenBank GI number and a Molecular Function GO slim term.
(ZIP)

S7 File Fully screened compound vs target cluster binary matrix. This is a zipped Excel readable tab separated text file with PubChem compound ids (cid) for each compound in the first column. The first (header) line contains a unique representative UniProt identifier for each sequence-similar protein target cluster. Six targets had no UniProt translation and include a GenBank GI number instead, prefixed with “gi_”. Zero values represent inactive compound-target activity outcomes, while values of one represent active outcomes.
(ZIP)

S8 File Pfam domain co-occurrence on protein targets. This file reports all protein domain combinations that occur together on the same protein targets among the PubChem BioAssay target set, as identified with the HMMER analysis described in the main text. This is a zipped Excel readable tab separated text file with Pfam domain ids in the first column, and domain compositions including this Pfam domain in the second column with domains separated by an underscore. The third column lists all domain co-occurrences (also underscore separated) for this Pfam domain, and is repeated (and identical) for all rows corresponding to the same Pfam domain. Out of 2798 Pfam domains which occur on multiple-target proteins, approximately half (1388 domains) is found in only one unique combination on these targets.
(ZIP)

## References

[1] Kim S, Thiessen PA, Bolton EE, Chen J, Fu G, Gindulyte A, et al PubChem Substance and Compound databases. Nucleic Acids Res. 2016 1;44(D1):D1202–D1213. 10.1093/nar/gkv951 26400175 PMC4702940

[2] Harrow J, Frankish A, Gonzalez JM, Tapanari E, Diekhans M, Kokocinski F, et al GENCODE: The reference human genome annotation for The ENCODE Project. Genome Res. 2012 9;22(9):1760–1774. 10.1101/gr.135350.111 22955987 PMC3431492

[3] Wang Y, Suzek T, Zhang J, Wang J, He S, Cheng T, et al PubChem BioAssay: 2014 update. Nucleic Acids Res. 2013 12;42(D1):D1075–D1082. 10.1093/nar/gkt978 24198245 PMC3965008

[4] Gaulton A, Bellis LJ, Bento AP, Chambers J, Davies M, Hersey A, et al ChEMBL: a large-scale bioactivity database for drug discovery. Nucleic Acids Res. 2012 1;40(Database issue):D1100–7. 10.1093/nar/gkr777 21948594 PMC3245175

[5] Keiser MJ, Roth BL, Armbruster BN, Ernsberger P, Irwin JJ, Shoichet BK. Relating protein pharmacology by ligand chemistry. Nat Biotechnol. 2007 2;25(2):197–206. 10.1038/nbt1284 17287757

[6] Yan SF, King FJ, He Y, Caldwell JS, Zhou Y. Learning from the Data: Mining of Large High-Throughput Screening Databases. J Chem Inf Model. 2006 11;46(6):2381–2395. 10.1021/ci060102u 17125181

[7] Wassermann AM, Peltason L, Bajorath J. Computational Analysis of Multi-target Structure-Activity Relationships to Derive Preference Orders for Chemical Modifications toward Target Selectivity. ChemMedChem. 2010 4;5(6):847–858. 10.1002/cmdc.201000064 20414918

[8] Han L, Wang Y, Bryant SH. A survey of across-target bioactivity results of small molecules in PubChem. Bioinformatics. 2009 8;25(17):2251–2255. 10.1093/bioinformatics/btp380 19549631 PMC2734317

[9] Cheng T, Wang Y, Bryant SH. Investigating the correlations among the chemical structures, bioactivity profiles and molecular targets of small molecules. Bioinformatics. 2010 11;26(22):2881–2888. 10.1093/bioinformatics/btq550 20947527 PMC2971579

[10] Wang Y, Xiao J, Suzek TO, Zhang J, Wang J, Zhou Z, et al PubChem’s BioAssay Database. Nucleic Acids Res. 2011 12;40(D1):D400–D412. 10.1093/nar/gkr1132 22140110 PMC3245056

[11] Szklarczyk D, Santos A, von Mering C, Jensen LJ, Bork P, Kuhn M. STITCH 5: augmenting protein-chemical interaction networks with tissue and affinity data. Nucleic Acids Res. 2016;44(D1):D380–4. 10.1093/nar/gkv1277 26590256 PMC4702904

[12] McGovern SL, Helfand BT, Feng B, Shoichet BK. A Specific Mechanism of Nonspecific Inhibition. J Med Chem. 2003 9;46(20):4265–4272. 10.1021/jm030266r 13678405

[13] Schmidt U, Struck S, Gruening B, Hossbach J, Jaeger IS, Parol R, et al SuperToxic: a comprehensive database of toxic compounds. Nucleic Acids Res. 2009 1;37(Database):D295–D299. 10.1093/nar/gkn850 19004875 PMC2686515

[14] Kuhn M, Campillos M, Letunic I, Jensen LJ, Bork P. A side effect resource to capture phenotypic effects of drugs. Mol Syst Biol. 2010;6:343 10.1038/msb.2009.98 20087340 PMC2824526

[15] Lounkine E, Keiser MJ, Whitebread S, Mikhailov D, Hamon J, Jenkins JL, et al Large-scale prediction and testing of drug activity on side-effect targets. Nature. 2012 6;486(7403):361–367. 10.1038/nature11159 22722194 PMC3383642

[16] Irwin JJ, Sterling T, Mysinger MM, Bolstad ES, Coleman RG. ZINC: A Free Tool to Discover Chemistry for Biology. J Chem Inf Model. 2012 7;52(7):1757–1768. 10.1021/ci3001277 22587354 PMC3402020

[17] Moya-Garcia AA, Ranea JAG. Insights into polypharmacology from drug-domain associations. Bioinformatics. 2013 7;29(16):1934–1937. 10.1093/bioinformatics/btt321 23740740

[18] Griffith M, Griffith OL, Coffman AC, Weible JV, McMichael JF, Spies NC, et al DGIdb: mining the druggable genome. Nat Methods. 2013 10;10(12):1209–1210. 10.1038/nmeth.2689 24122041 PMC3851581

[19] Kumar RD, Chang LW, Ellis MJ, Bose R. Prioritizing Potentially Druggable Mutations with dGene: An Annotation Tool for Cancer Genome Sequencing Data. PLoS ONE. 2013 6;8(6):e67980 10.1371/journal.pone.0067980 23826350 PMC3694871

[20] Hewett M. PharmGKB: the Pharmacogenetics Knowledge Base. Nucleic Acids Res. 2002 1;30(1):163–165. 10.1093/nar/30.1.163 11752281 PMC99138

[21] Dandapani S, Marcaurelle LA. Grand Challenge Commentary: Accessing new chemical space for’undruggable’ targets. Nat Chem Biol. 2010 12;6(12):861–863. 10.1038/nchembio.479 21079589

[22] Zerhouni E. The NIH Roadmap. Science. 2003 10;302(5642):63–72. 10.1126/science.1091867 14526066

[23] Zhang J, Lushington GH, Huan J. Characterizing the Diversity and Biological Relevance of the MLPCN Assay Manifold and Screening Set. J Chem Inf Model. 2011 6;51(6):1205–1215. 10.1021/ci1003015 21568288 PMC3152445

[24] Hu Y, Bajorath J. High-resolution view of compound promiscuity. F1000Res. 2013 Jul;. 10.12688/f1000research.2-144.v1 PMC3799544 24358872

[25] Wishart DS, Knox C, Guo AC, Cheng D, Shrivastava S, Tzur D, et al DrugBank: a knowledgebase for drugs, drug actions and drug targets. Nucleic Acids Res. 2007 12;36(Database):D901–D906. 10.1093/nar/gkm958 18048412 PMC2238889

[26] Jasial S, Hu Y, Bajorath J. Determining the Degree of Promiscuity of Extensively Assayed Compounds. PLoS ONE. 2016 4;11(4):e0153873 10.1371/journal.pone.0153873 27082988 PMC4833426

[27] Dan ik V, Carrel H, Bodycombe NE, Seiler KP, Fomina-Yadlin D, Kubicek ST, et al Connecting Small Molecules with Similar Assay Performance Profiles Leads to New Biological Hypotheses. J Biomol Screening. 2014 5;19(5):771–781. 10.1177/1087057113520226 PMC5554958 24464433

[28] Baell JB, Holloway GA. New Substructure Filters for Removal of Pan Assay Interference Compounds (PAINS) from Screening Libraries and for Their Exclusion in Bioassays. J Med Chem. 2010 4;53(7):2719–2740. 10.1021/jm901137j 20131845

[29] Visser U, Abeyruwan S, Vempati U, Smith RP, Lemmon V, Schürer SC. BioAssay Ontology (BAO): a semantic description of bioassays and high-throughput screening results. BMC Bioinf. 2011;12(1):257 10.1186/1471-2105-12-257 PMC3149580 21702939

[30] Howe EA, de Souza A, Lahr DL, Chatwin S, Montgomery P, Alexander BR, et al BioAssay Research Database (BARD): chemical biology and probe-development enabled by structured metadata and result types. Nucleic Acids Res. 2015 1;43(D1):D1163–D1170. 10.1093/nar/gku1244 25477388 PMC4383997

[31] Malo N, Hanley JA, Cerquozzi S, Pelletier J, Nadon R. Statistical practice in high-throughput screening data analysis. Nat Biotechnol. 2006 2;24(2):167–175. 10.1038/nbt1186 16465162

[32] Punta M, Coggill PC, Eberhardt RY, Mistry J, Tate J, Boursnell C, et al The Pfam protein families database. Nucleic Acids Res. 2011 12;40(D1):D290–D301. 10.1093/nar/gkr1065 22127870 PMC3245129

[33] Pruitt KD, Tatusova T, Brown GR, Maglott DR. NCBI Reference Sequences (RefSeq): current status, new features and genome annotation policy. Nucleic Acids Res. 2011 12;40(D1):D130–D135. 10.1093/nar/gkr1079 22121212 PMC3245008

[34] Hauser M, Mayer CE, Söding J. kClust: fast and sensitive clustering of large protein sequence databases. BMC Bioinf. 2013;14(1):248 10.1186/1471-2105-14-248 PMC3843501 23945046

[35] Backman TWH, Girke T. bioassayR: Cross-Target Analysis of Small Molecule Bioactivity. J Chem Inf Model. 2016 7;0(ja):null. 10.1021/acs.jcim.6b00109 PMC5330305 27367556

[36] Jalencas X, Mestres J. On the origins of drug polypharmacology. Med Chem Commun. 2012 12;4(1):80–87. 10.1039/C2MD20242E

[37] The Gene Ontology Consortium. Gene Ontology Consortium: going forward. Nucleic Acids Res. 2015 1;43(D1):D1049–D1056. 10.1093/nar/gku1179 25428369 PMC4383973

[38] Laherrère J, Sornette D. Stretched exponential distributions in nature and economy: “fat tails” with characteristic scales. Eur Phys J B. 1998 5;2(4):525–539. 10.1007/s100510050276

[39] Shun J Lee JYW. Exploiting the promiscuity of imatinib. Journal of Biology. 2009;8(3):30 10.1186/jbiol134 19435483 PMC2689438

[40] Knight ZA, Lin H, Shokat KM. Targeting the cancer kinome through polypharmacology. Nature reviews Cancer. 2010 2;10(2):130–137. 10.1038/nrc2787 20094047 PMC2880454

[41] Feng BY, Simeonov A, Jadhav A, Babaoglu K, Inglese J, Shoichet BK, et al A High-Throughput Screen for Aggregation-Based Inhibition in a Large Compound Library. J Med Chem. 2007 5;50(10):2385–2390. 10.1021/jm061317y 17447748

[42] Uitert Mv, Meuleman W, Wessels L. Biclustering Sparse Binary Genomic Data. J Comput Biol. 2008 12;15(10):1329–1345. 10.1089/cmb.2008.0066 19040367

[43] Cao Y, Charisi A, Cheng LC, Jiang T, Girke T. ChemmineR: a compound mining framework for R. Bioinformatics. 2008 7;24(15):1733–1734. 10.1093/bioinformatics/btn307 18596077 PMC2638865

[44] Yıldırım MA, Goh KI, Cusick ME, Barabási AL. Drug—target network. Nature. 2007;25(10):1119–1126. 10.1038/nbt1338 17921997

[45] Eddy SR. A new generation of homology search tools based on probabilistic inference. Genome Inform. 2009 10;23(1):205–211. 20180275

[46] Consortium TU. Reorganizing the protein space at the Universal Protein Resource (UniProt). Nucleic Acids Res. 2011 11;40(D1):gkr981–D75. 10.1093/nar/gkr981 PMC3245120 22102590

[47] Mitchell A, Chang HY, Daugherty L, Fraser M, Hunter S, Lopez R, et al The InterPro protein families database: the classification resource after 15 years. Nucleic Acids Res. 2015 1;43(Database issue):D213–21. 10.1093/nar/gku1243 25428371 PMC4383996

[48] Falcon S, Gentleman R. Using GOstats to test gene lists for GO term association. Bioinformatics. 2007 1;23(2):257–258. 10.1093/bioinformatics/btl567 17098774

[49] Saubern S, Guha R, Baell JB. KNIME Workflow to Assess PAINS Filters in SMARTS Format. Comparison of RDKit and Indigo Cheminformatics Libraries. Molecular Informatics. 2011 10;30(10):847–850. 10.1002/minf.201100076 27468104

[50] Csardi G, Nepusz T. The igraph software package for complex network research. InterJournal. 2006;.

[51] Bastian M, Heymann S, Jacomy M. Gephi: an open source software for exploring and manipulating networks. ICWSM. 2009;.

[52] Jacomy M, Venturini T, Heymann S, Bastian M. ForceAtlas2, a continuous graph layout algorithm for handy network visualization designed for the Gephi software. PLoS ONE. 2014;9(6):e98679 10.1371/journal.pone.0098679 24914678 PMC4051631

[53] Urban JD, Clarke WP, von Zastrow M, Nichols DE, Kobilka B, Weinstein H, et al Functional Selectivity and Classical Concepts of Quantitative Pharmacology. J Pharmacol Exp Ther. 2006 6;320(1):1–13. 10.1124/jpet.106.104463 16803859

[54] Kenakin T. Functional selectivity and biased receptor signaling. J Pharmacol Exp Ther. 2011 2;336(2):296–302. 10.1124/jpet.110.173948 21030484

[55] Lipinski CA, Lombardo F, Dominy BW, Feeney PJ. Experimental and computational approaches to estimate solubility and permeability in drug discovery and development settings. Adv Drug Deliv Rev. 2001 3;46(1–3):3–26. 10.1016/S0169-409X(00)00129-0 11259830

[56] Lehár J, Krueger AS, Avery W, Heilbut AM, Johansen LM, Price ER, et al Synergistic drug combinations tend to improve therapeutically relevant selectivity. Nat Rev Drug Discovery. 2009 7;27(7):659–666. 10.1038/nbt.1549 PMC2708317 19581876

[57] Muller PY, Milton MN. The determination and interpretation of the therapeutic index in drug development. Nat Rev Drug Discovery. 2012 8;11(10):751–761. 10.1038/nrd3801 22935759

