# Supplementary material for: Large-scale bioactivity analysis of the small-molecule assayed proteome
Source: PLoS One. 2017 Feb 8;12(2):e0171413. doi: 10.1371/journal.pone.0171413 (PMC5298297; doi:10.1371/journal.pone.0171413)
Supplement: S1 Text — This text contains more analysis details on the fully screened sub-matrix we provide as a downloadable reference, an algebraic estimate of error rates, our target selectivity by molecular size analysis, and a more in-depth discussion and methods for the stretched exponential selectivity distribution. (PDF) [file pone.0171413.s001.pdf]

Supporting Information: S1 Text
Large-scale Bioactivity Analysis of the Druggable Proteome

Tyler William H Backman[1,2], Daniel S Evans[3], Thomas Girke[2*]

1 Department of Bioengineering, University of California Riverside, Riverside, California, United States of America
2 Institute for Integrative Genome Biology, University of California Riverside, Riverside, California, United States of America
3 California Pacific Medical Center Research Institute, San Francisco, California, United States of America

* thomas.girke@ucr.edu

Target Selectivity Distribution

	Median Selectivity			Mean Selectivity			Trimmed Mean Selectivity		
Compounds	Target	Cluster	Domain	Target	Cluster	Domain	Target	Cluster	Domain
FDA Approved	7	6	4	13.7 ± 19.6	10.6 ± 14.3	5.8 ± 6.5	6.9 ± 5.1	6.4 ± 5.0	4.9 ± 5.2
Non-FDA	2	2	2	4.1 ± 5.8	4.0 ± 5.4	3.5 ± 4.1	3.5 ± 3.5	3.4 ± 3.4	3.2 ± 3.2

Table I. Distribution of active proteins per compound. Selectivity is quantified by three methods which cluster protein targets across increasingly large evolutionary distances, as described in the text. Mean values include a standard deviation after the $\pm$ symbol. Trimmed mean represents the mean of compounds with values of 20 or fewer. We include a trimmed mean, as mean selectivities are highly skewed by a small number of highly promiscuous compounds, whose selectivity counts reflect the screening data volume rather than the bioactivity profiles.

Fully Screened Sub-Matrix

As the assay participation for individual compounds is heavily biased, with both very highly screened and infrequently screened compounds (Table 1 and S3 Fig), we sought to identify the largest fully screened sub-matrix of compounds and targets within these data. The size of this sub-matrix quantifies the magnitude of screening bias towards a specific region of the compound-target space, and can also serve as a computational resource for bioactivity analysis methods which cannot accommodate a sparse matrix. Identifying this sub-matrix is a complex nonlinear optimization problem, and simply taking the combination of the most highly screened compounds and targets will not reliably identify the largest bicluster. We chose the BicBin algorithm (set to find non-sparse clusters) for practical reasons, as it scales to large matricies, and finds top-scoring clusters first [1]. As such, we used the BicBin biclustering algorithm to identify the largest fully screened sub-matrix with at least one active outcome in each row and column within the clustered compound-target matrix, as described in the "Fully Screened Sub-Matrix Methods" section of this document. The resulting fully screened matrix had dimensions of 65204 compounds by 260 target clusters, where 23 of the compounds are FDA approved drugs. This number of compounds represents the size of the intersection among the large compound libraries used across many large-scale

screening experiments. For example, at the time of writing, PubChem BioAssay contains 128 protein target assays deposited by the NIH Chemical Genomics Center (NCGC) high-throughput screening center that each contain over 70000 compounds. This matrix had an overall activity density (fraction of active scores) of 0.94%, which is very close to the fraction of actives in the full set of PubChem BioAssay protein target data.

This sub-matrix is useful as a representative data set for many data mining techniques. For example, patterns of target selectivity, and compound-target network connectivity can be investigated without introducing bias from varying compound assay participation as is present in the full PubChem BioAssay data. This reference can also be used to train and cross-validate machine learning and imputation methods, in order to assess there performance at imputing missing values from a sparse bioactivity matrix. Lastly, it can be used as a reference for designing custom reduced dimensionality high throughput screening bioactivity fingerprints (HTSFPs), *e.g.* with Principal Component Analysis that still encode a defined portion of the overall variation in bioactivity profiles present in the larger set of public bioactivity data. This matrix is provided as a downloadable resource in the S7 File of Supporting Information, however the full (sparse) bioactivity matrix is used for all analysis in this study, in order to make use of as much relevant data as possible.

Fully Screened Sub-Matrix Methods

We used the BicBin biclustering algorithm to identify the largest fully screened sub-matrix from the clustered compound-target matrix described in the main text, where all values are represented as conclusively active or inactive. We then removed all rows and columns without at least one active experimental outcome. The options $\alpha = 0.5$ and $\beta = 0.8$ were used to bias the result towards including more protein targets, such that the resulting bicluster would include multiple target clusters, instead of summarizing only one or a small number of extremely large assays. We ran the BicBin algorithm enough times to reliably converge on a stable best-scoring bicluster.

Error Rate Estimate

For those compound-target pairs screened 2-4 times, the quantity of agreement among replicates is shown in Table II. We quantify the number of times replicated sets are all active, all inactive, or have varying levels of disagreement. The error rate in these data was estimated from the pair data (compound-target pairs screened in exactly two assays) by solving the system of equations I, II, and III below. As the results can be represented by three independent equations, we can solve for three unknowns. In this case we solve algebraically for the number of true positives, true negatives, and error rate. This analysis makes the simplification that the false positive and false negative error rates are identical, as additional information such as a gold standard reference would be necessary to compute these separately.

The count of double inactives shown in Equation I represents the sum of two truly inactive results, plus the number of double false negatives. Here p is the number of true positives, n is the number of true negatives, e is the error rate, I_1 is the set of result pairs where the first was inactive, and I_2 is the set were the second is inactive. I_1^c is the set of result pairs where the first was active, and I_2^c is the set were the second is active. The order of I_1 and I_2 is irrelevant. The count of single inactives shown in Equation II represents the sum of inactive pairs with one false positive, and active pairs with one false negative. The count of zero inactives shown in Equation III represents the sum of two truly active results, plus the number of double false positives.

Times Screened	0 Inactives	1 Inactive	2 Inactives	3 inactives	4 inactives
2	181758	292274	20746238		
3	22740	34868	92323	3158813	
4	8510	11922	20328	31672	654268

Table II. Disagreement and agreement among compound target pairs screened 2-4 distinct times. Shown are the number of pairs that had a given number of inactive (vs active) results in these replicates. For example if a given protein target pair was screened in two assays (Times Screened = 2) and both results were inactive it's count would be added to the "2 Inactives" column. If the two assays disagree and one showed active and the other inactive, it is counted in the "1 Inactive" column.

$$\overbrace{|I_1 \cap I_2|}^{\text{two inactives count}} = \overbrace{n(1-e^2)}^{\text{double true negatives}} + \overbrace{pe^2}^{\text{double false negatives}} \quad \text{(I)}$$

$$\overbrace{|I_1 \triangle I_2|}^{\text{one inactive count}} = \overbrace{2ne}^{\text{one false positive}} + \overbrace{2pe}^{\text{one false negative}} \quad \text{(II)}$$

$$\overbrace{|I_1^{\mathsf{c}} \cap I_2^{\mathsf{c}}|}^{\text{zero inactives count}} = \overbrace{p(1-e^2)}^{\text{double true positives}} + \overbrace{ne^2}^{\text{double false positives}} \quad \text{(III)}$$

Solving for e based on the data in the first row of Table II yields an estimated error rate of approximately $e = 0.00698$, or roughly 0.7% with approximately p =181k true positives, and approximately n =20.7M true negatives. If the false positive and false negative rates were identical, this would represent a fraction of true active compound-target outcomes of about 0.86%, which is somewhat lower than the 1.3% of values which are active in the entire PubChem BioAssay protein target data we analyze in this study. If we additionally make the approximation that the fraction of true positives in this replicated data is close to that in the larger non-replicated set, we estimate that the overall fraction of active results which are true positives is roughly $100 * 0.86/1.3 = 66\%$, ignoring the negligible false negatives. This error rate is low enough that only a small portion (roughly 0.56%) of the double positive (0 inactives) results are likely to be double false positives rather than true positives, while most of the single inactive results among replicated pairs are a result of false positives.

Target Selectivity by Molecular Size

To investigate the possibility of a relationship between target selectivity and molecular size, we quantified the distribution of molecular sizes for compounds with different numbers of distinct sequence active targets, *i.e.* cluster selectivity. The definition of molecular size used here is the quantity of non-hydrogen atoms. We analyzed these data to identify both general trends of selectivity in relation to molecular size, and investigated distinct patterns among the largest and smallest compounds. All highly screened compounds with 1 to 10 active targets are shown in S4 Fig. Many of the very largest compounds in PubChem BioAssay with over 300 heavy atoms were screened in a large number of assays but reported inconclusive results in most or all, and were excluded here. This suggests that there may be technical limitations to these assay methodologies with regard to extremely large compounds. Non-FDA compounds have a slightly higher mean molecular size than FDA approved drugs (Table III and S4 Fig). While the overall distribution of molecular size is roughly the same across compounds with differing numbers of active targets, the largest and smallest compounds show

greater target selectivity (fewer targets) than non-FDA compounds, as can be seen in the tail lengths of S4 Fig. While these compounds vary in size from 1 to 302 heavy atoms, all compounds with a size under 3 or over 190 (a total of 4 and 8 respectively) have 3 or fewer active targets. Compounds with greater than 10 active protein targets show approximately the same distribution of size shown here for 7-10 active targets, and were excluded from the plot due to space considerations.

Compounds	Mean±SD Size	Size Range
FDA Approved	23.0 ± 9.3	$4 - 87$
Other	26.3 ± 7.0	$2 - 302$

Table III. Variation in molecular size for FDA approved and non-FDA compounds. FDA approved compounds have a slightly lower mean size than non-FDA compounds. Only highly screened active compounds were included in this calculation.

The target selectivity of the 40 largest highly screened active FDA approved drugs is shown in Table IV, along with the common name and therapeutic utility (annotation from DrugBank). The majority of these compounds are either natural products or semi-synthetic drugs derived from natural products. It has been reported in previous literature that approximately half of the naturally discovered drugs violate the Lipinski Rule of Five in terms of molecular size, and number of rotatable bonds, despite the fact that effective and bioavailable synthetic drugs rarely violate these rules [2, 3]. As discussed by Ganesan, this may in part be due to naturally evolved synthetic processes that allow large molecules to maintain low hydrophobicity and intermolecular H-bond donating potential, as well as the ability of natural products to mimic endogenous metabolites allowing them to utilize active transport systems [2]. Overall this subset shows activity against a greater number of targets than most FDA approved compounds (median 12.5 vs 7 for all drugs), however there is substantial variation by drug class. Several of the most selective large molecules such as streptomycin and amikacin are natural antibiotics evolved to specifically inhibit prokaryotic ribosomal RNA structures, which are not among the screening targets in our analysis [4, 5]. Many of the least selective large molecules are highly promiscuous cytotoxic compounds (such as Suramin) which have dual utility as cancer chemotherapy agents and as antiparasitic drugs [6–8].

Stretched Exponential Selectivity Distribution

As a curvature can be seen in the semi-log plot space for each of the target selectivity distributions shown in S5 Fig, these data have a regular pattern that cannot be described fully by an exponential probability mass distribution. To model the distribution of target selectivity we fit the distinct sequence target data to three probability density function models, the exponential (equation IV), power law (equation V), and stretched exponential function (equation VI) where x represents the number of active targets, and each function includes two fit parameters. While the number of active targets for each compound is a discrete value suggesting a probability mass function, here we fit a continuous function to approximate it. The exponential and power law functions fit with roughly opposite sign residuals ($R^2 = 0.99131$ and $R^2 = 0.97916$ respectively), and the stretched exponential, which is a composite of the other two, fit the data closely ($R^2 = 0.99912$) as shown in S5 Fig.

$$P(x) = fe^{-(\frac{x}{g})} \tag{IV}$$

$$P(x) = ax^b \tag{V}$$

Name	PubChem CID	Size	Active Targets	Tested Targets	Description
Cisatracurium Besylate	62886	87	1	59	muscle relaxant
Suramin	5361	86	22	63	antiparasitic
Amphotericin B	5280965	65	3	71	antifungal
Paclitaxel	36314	62	20	293	antimitotic
Rifabutin	6323490	61	9	228	antibiotic
Vincristine	5978	60	17	57	antimitotic
Vinblastine	241903	59	4	25	antimitotic
Docetaxel	148124	58	9	33	antimitotic
Rifaximin	6436173	57	2	15	antibiotic
Digitoxin	441207	54	28	229	cardiac glycoside
Erythromycin	12560	51	19	330	antibiotic
Posaconazole	147912	51	5	28	antifungal
Atazanavir	148192	51	6	36	antiretroviral protease inhibitor
Ritonavir	392622	50	26	243	antiretroviral protease inhibitor
Itraconazole	55283	49	15	34	antifungal
Lopinavir	92727	46	14	76	antiretroviral protease inhibitor
Rescinnamine	5280954	46	10	260	antihypertensive
Tubocurarine	6000	45	2	61	neuromuscular blocker
Lercanidipine	65866	45	3	39	antihypertensive
Indinavir	5362440	45	10	10	antiretroviral protease inhibitor
Reserpine	5770	44	28	339	antipsychotic
Ergotamine	8223	43	38	42	vasoconstrictor
Dihydroergotamine	10531	43	31	88	vasoconstrictor
Bromocriptine	31101	43	37	77	dopamine agonist
Irinotecan	60838	43	11	22	antimitotic
Nonoxynol-9	72385	43	1	44	surfactant
Benzonatate	7699	42	2	322	antitussive
Deserpidine	8550	42	11	24	antihypertensive
Etoposide	36462	42	22	273	antimitotic
Zafirlukast	5717	41	42	229	leukotriene receptor antagonist
Ouabain	439501	41	19	298	cardiac glycoside
Montelukast	5281040	41	25	28	leukotriene receptor antagonist
Hexafluronium	9434	40	3	14	neuromuscular blocker
Streptomycin	19649	40	1	34	antibiotic
Amikacin	37768	40	1	43	antibiotic
Nelfinavir	64143	40	27	321	antiretroviral protease inhibitor
Lapatinib	208908	40	17	166	tyrosine kinase inhibitor
Deferoxamine	2973	39	5	182	chelating agent
Doxorubicin	31703	39	30	43	antitumor antibiotic
Telmisartan	65999	39	15	289	antihypertensive

Table IV. Target selectivity of the 40 largest highly screened active FDA Approved Drugs. Sorted by decreasing size (non-hydrogen atom count). The description column represents one common clinical use of each compound, but may not represent its only known therapeutic utility.

$$P(x) = e^{-(\frac{x}{x_0})^c} \tag{VI}$$

The stretched exponential fit the non-FDA cluster selectivity distribution for 1-20 targets with best fit parameters $c = 0.6423$ and $x_0 = 0.9487$. Fitting the FDA approved drug data to the stretched exponential (equation VI, $R^2 = 0.9033$) yielded best fit parameters $c = 0.2445$ and $x_0 = 0.06681$. The FDA approved distribution was fit

slightly better by an exponential distribution, and exhibits much less curvature in semi-log space. The fact that the exponential function fits relatively well suggests that the distribution of active targets for each compound approximately follows an exponential underlying process. That is, each compound-target interaction can be seen as an independent event of roughly equal probability. However, if these were singular independent outcomes with the same probability we would expect the exponential function to fit better than the stretched exponential, but as shown that is not the case for the non-FDA compounds.

Stretched exponential probability distributions have been documented in a large number of natural and artificial processes, many of which have been shown to follow this probability distribution due to an underlying multiplicative process, where a series of chained events, each with different probabilities is involved in the resulting outcome quantified [9–12]. For example, they can be observed in the distribution of binding times for single molecule enzyme-substrate combinations, and protein structural relaxation times [13–17]. This lends a possible physical interpretation to the two fit parameters in these processes. The value of the exponent, c describes the curvature in semilog space, and arises from the underlying multiplicity of the process. In a stretched exponential, the exponent c is always smaller than one, with a value inverse to the number of generations, $m = \frac{1}{c}$ in the underlying multiplicative cascade [9]. Further investigation will be necessary to elucidate the nature of the possible key underlying multiplicative steps involved in compound-target interactions suggested by this analysis.

Stretched Exponential Selectivity Distribution Methods

Numeric fit was computed with the *nls* (Nonlinear Least Squares) function in the R programming language. Each function included two fit parameters.

Target Selectivity Distribution Among Targets Sharing a Common Pfam Domain

To investigate whether the higher promiscuity of FDA approved compounds for related targets is due to more extensive testing within target clusters, we quantified the target selectivity of FDA approved and non-FDA approved compounds separately across targets sharing a common Pfam domain. Out of the 2838 Pfam domains with active compounds represented in PubChem BioAssay, 57 Pfam domains contained sufficient screening volume to meet our minimum requirements for this investigation. These requirements included at least 10 distinct protein targets within each domain cluster as well as bioassay data of FDA approved compounds for at least 10 targets.

For each of these 57 domains, we computed the number of screened and active targets for FDA approved and non-FDA approved active compounds. To create a similar screening distribution for each domain cluster, we considered only compounds tested against at least 10 different targets annotated with the same domain, and active against at least one of these. Within these 57 domains, 20 domains had at least 10 highly screened FDA approved drugs meeting this criteria, and therefore are strong candidates to contribute to the higher target promiscuity among FDA approved compounds.

Out of these 20 domains, 19 domains had a higher median number of screened targets among FDA approved compounds, and 1 domain had a higher median number of screened targets among non-FDA compounds. Among the active targets, 9 domains had a higher median number of active targets among FDA approved compounds, 10 domains had an equal median number between the FDA and non-FDA compounds, and 1 domain had a higher median number of active targets among non-FDA compounds.

Out of these 20 domains, many tend to co-occur on the same proteins, and therefore represent largely the same screening data. Therefore, we chose a single representative

domain for each co-occurrence set as described in the "De-duplication of Single Domain Clusters" methods section of the main text. This narrowed down the data to just 8 representative Pfam domains. For these 8 domains, we plot the distribution of active and screened targets in S7 Fig of Supporting Information panel A and B respectively. Table V lists the number of highly screened FDA approved compounds, non-FDA compounds, and total number of protein targets for each of these domains.

As shown in S7 Fig panel A, for most of these domains the number of active targets is much greater for FDA approved compounds, than for non-FDA compounds, however direct comparison is hindered by both sets not having an identical number of screened targets. To control for this difference, within each domain we iteratively and randomly excluded a single activity outcome from the most highly screened FDA approved drug in each iteration, until the median number of screened targets for the FDA approved drugs was equal to, or slightly lower than the non-FDA compounds. After this exclusion process, out of the previously mentioned 20 domains, 8 domains still had a greater median number of active targets among the FDA approved compounds, 11 domains had an equal median number, and only 1 domain had a greater median number of active targets among the non-FDA compounds. For the 8 representative domains for each co-occurrence set mentioned above, we plot the distribution of active and screened targets in S7 Fig of Supporting Information panel C and D respectively, after the iterative random removal of activity outcomes. As shown in S7 Fig panel C, the greater target promiscuity across proteins sharing a single domain remains similar to panel A, even after controlling for differences in assay participation. The individual domains which show high target cross-reactivity among the FDA approved drugs, are also largely the same domains we found enriched in the largest drug-target biclusters (see Table 7 in the main text). The largest cross-reactivity occurs across the targets which contain the domain PF00001 7 transmembrane receptor characteristic of rhodopsin-like GPCRs, which is expected, as it is the most common target class for the FDA approved drugs. One possible explanation for the higher cross-reactivity among FDA approved compounds is that many of these drugs have been heavily optimized to bind to their targets with a high affinity. This higher affinity may result in more frequent cross-reactions with related targets, especially in high-throughput assays performed with a limited number of dosages.

Pfam Domain	FDA Approved Compounds	Non-FDA Compounds	Total Protein Targets
PF00001 7 transmembrane receptor (rhodopsin family)	247	80065	612
PF00069 Protein kinase domain	65	24228	602
PF14531 Kinase-like	47	18864	324
PF00104 Ligand-binding domain of nuclear hormone receptor	291	14048	128
PF00520 Ion transport protein	38	9634	124
PF00067 Cytochrome P450	40	18	70
PF13191 AAA ATPase domain	77	9099	54
PF00194 Eukaryotic-type carbonic anhydrase	23	157	23

Table V. Pfam protein domains with at least 10 screened protein targets, and at least 10 highly screened active FDA approved drugs. Domains are sorted decreasingly by number of screened proteins in PubChem BioAssay. The "FDA Approved Compounds" column shows the number of FDA approved compounds tested against at least 10 proteins annotated with this domain, and active against at least one. The "Non-FDA Compounds" column shows the total number of non-FDA approved compounds tested against at least 10 proteins annotated with this domain, and active against at least one. The "Total Protein Targets" shows the number of protein targets annotated with this domain, and having at least one active compound in the PubChem BioAssay data. For domains co-occurring on the same protein targets we kept one representative example, as described in the "De-duplication of Single Domain Clusters" methods section of the main text.

References

1. Uitert Mv, Meuleman W, Wessels L. Biclustering Sparse Binary Genomic Data. J Comput Biol. 2008 Dec;15(10):1329–1345.

2. Ganesan A. The impact of natural products upon modern drug discovery. Current Opinion in Chemical Biology. 2008 Jun;12(3):306–317.

3. Lipinski CA, Lombardo F, Dominy BW, Feeney PJ. Experimental and computational approaches to estimate solubility and permeability in drug discovery and development settings. Adv Drug Deliv Rev. 2001 Mar;46(1-3):3–26.

4. Böttger EC, Springer B, Prammananan T, Kidan Y, Sander P. Structural basis for selectivity and toxicity of ribosomal antibiotics. EMBO reports. 2001 Apr;2(4):318–323.

5. Carter AP, Clemons WM, Brodersen DE, Morgan-Warren RJ, Wimberly BT, Ramakrishnan V. Functional insights from the structure of the 30S ribosomal subunit and its interactions with antibiotics. Nature. 2000 Sep;407(6802):340–348.

6. Voogd TE, Vansterkenburg EL, Wilting J, Janssen LH. Recent research on the biological activity of suramin. Pharmacol Rev. 1993 Jun;45(2):177–203.

7. Dawson NA, Cooper MR, Figg WD, Headlee DJ, Thibault A, Bergan RC, et al. Antitumor activity of suramin in hormone-refractory prostate cancer controlling for hydrocortisone treatment and flutamide withdrawal as potentially confounding variables. Cancer. 1995 Aug;76(3):453–462.

8. Michel MC, Seifert R. Selectivity of pharmacological tools: implications for use in cell physiology. A Review in the Theme: Cell Signaling: Proteins, Pathways and Mechanisms. American Journal of Physiology - Cell Physiology. 2015 Apr;308(7):C505–C520.

9. Laherrère J, Sornette D. Stretched exponential distributions in nature and economy: "fat tails" with characteristic scales. Eur Phys J B. 1998 May;2(4):525–539.

10. Frisch U, Sornette D. Extreme Deviations and Applications. J Phys I France. 1997 Sep;7(9):1155–1171.

11. Naumis GG, Cocho G. The tails of rank-size distributions due to multiplicative processes: from power laws to stretched exponentials and beta-like functions. New Journal of Physics. 2007;9(8):286–286.

12. Klafter J, Shlesinger MF. On the relationship among three theories of relaxation in disordered systems. Proc Natl Acad Sci USA. 1986 Feb;83(4):848–851.

13. Fenimore PW, Frauenfelder H, McMahon BH, Young RD. Proteins are paradigms of stochastic complexity. Physica A: Statistical Mechanics and its Applications. 2005 Jun;351(1):1–13.

14. Flomenbom O, Velonia K, Loos D, Masuo S, Cotlet M, Engelborghs Y, et al. Stretched exponential decay and correlations in the catalytic activity of fluctuating single lipase molecules. Proc Natl Acad Sci USA. 2005 Feb;102(7):2368–2372.

15. Austin RH, Beeson KW, Eisenstein L. Dynamics of ligand binding to myoglobin. Biochemistry. 1975;14(24):5355–5373.

16. Lambright DG, Balasubramanian S, Boxer SG. Protein relaxation dynamics in human myoglobin. Chemical Physics. 1991 Dec;158(2-3):249–260.

17. Frauenfelder H, Wolynes PG, Austin RH. Biological Physics. Rev Mod Phys. 1999 Mar;71(2):S419–S430.
