# Supplementary figures and images for: Large-scale bioactivity analysis of the small-molecule assayed proteome
Source: PLoS One. 2017 Feb 8;12(2):e0171413. doi: 10.1371/journal.pone.0171413 (PMC5298297; doi:10.1371/journal.pone.0171413)

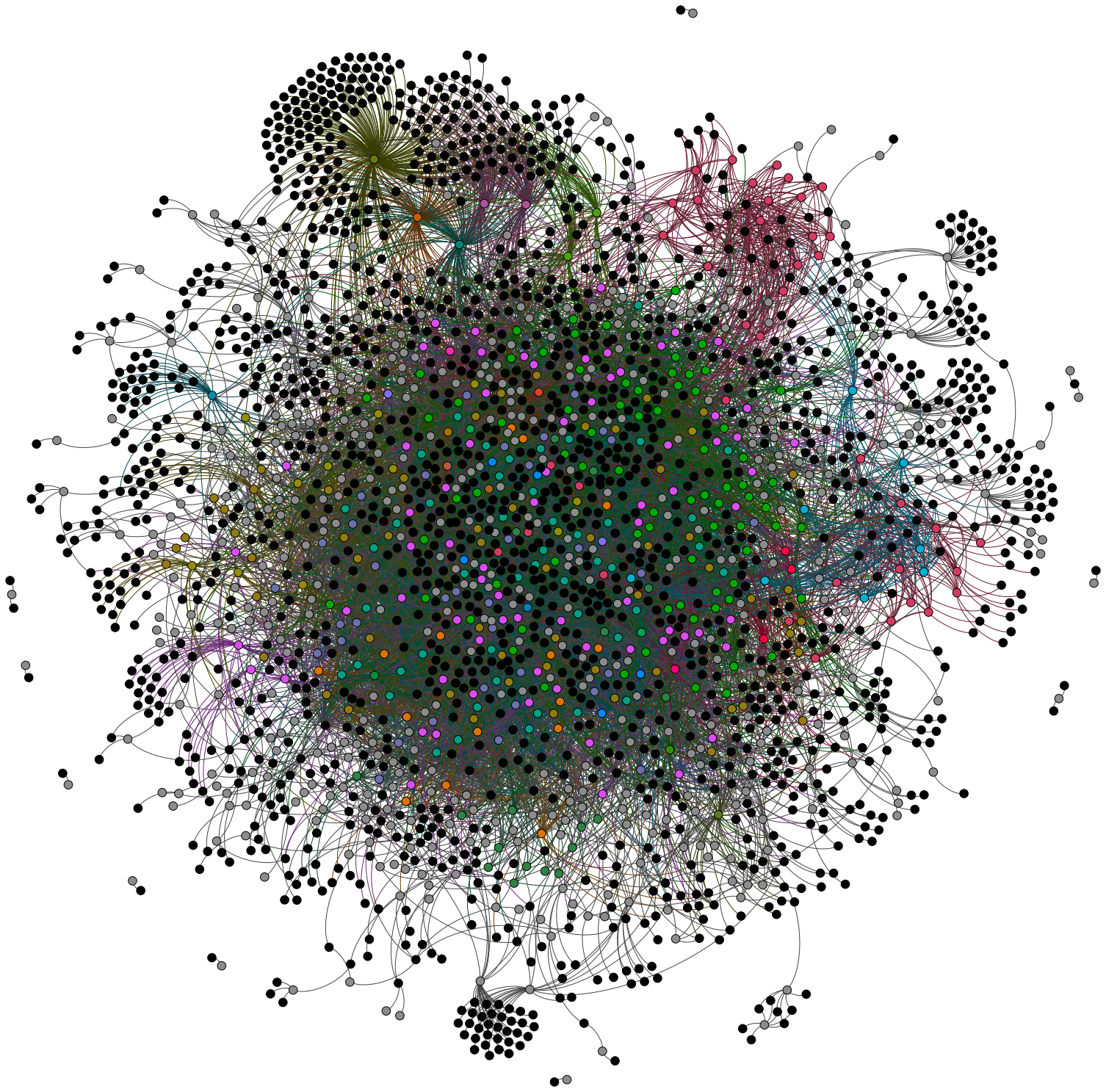

Supplement: S1 Fig — Protein targets are shown in black, with FDA approved drugs shown in color, based on their bioactivity bicluster. Unclustered compounds are shown in grey. No color key is provided, as some colors were reused in order to visualize a large number of biclusters. Node position is based on connectivity, with the same positions as in S2 Fig. (PDF) [file pone.0171413.s002.pdf]

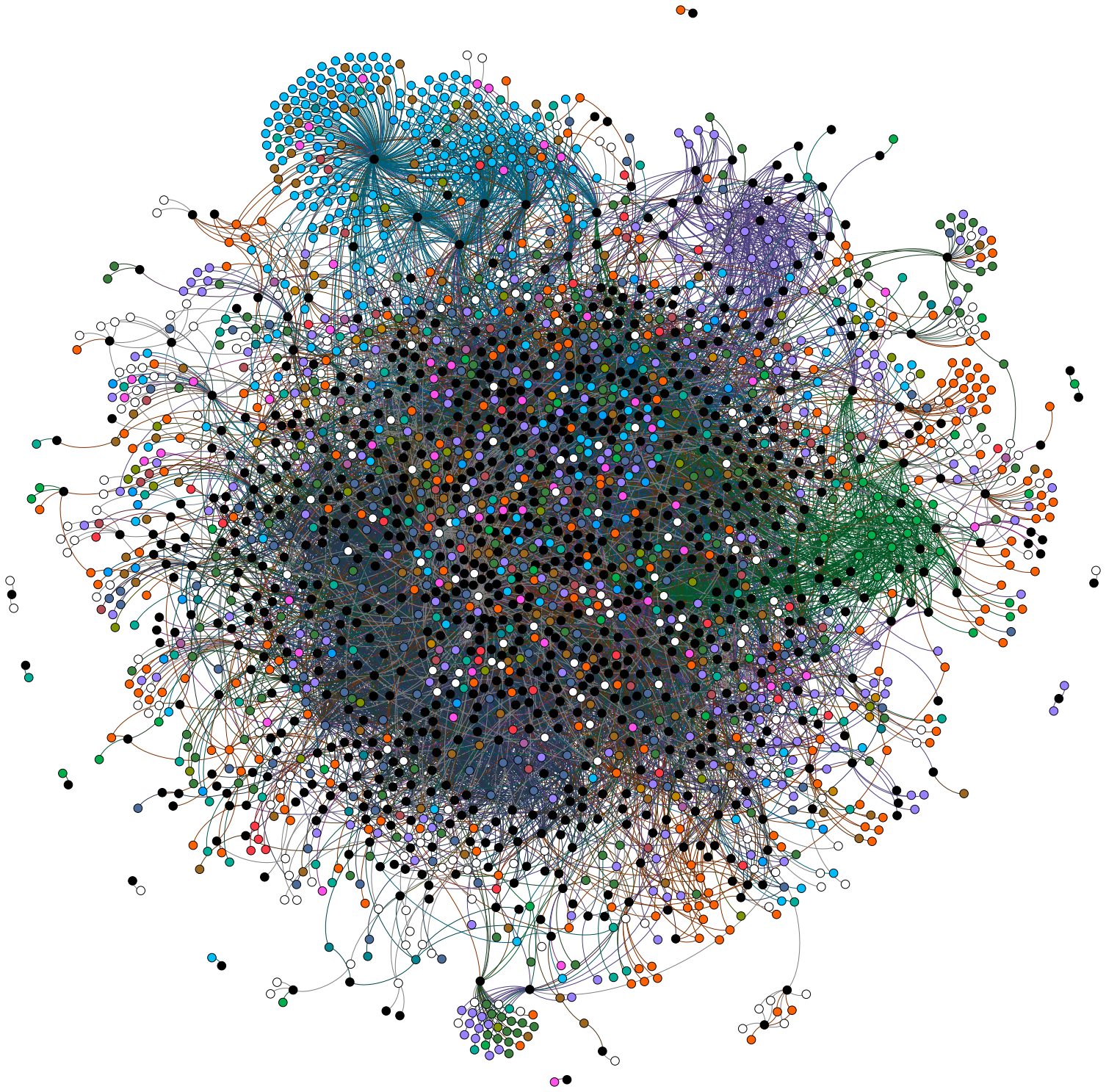

Supplement: S2 Fig — FDA approved drugs are shown in black, with protein targets show in color based on the most specific Molecular Function GO Slim term for each target. Unannotated targets are shown in white. No color key is provided, as some colors were reused in order to visualize a large number of GO terms. Node position is based on connectivity, with the same positions as in S1 Fig. (PDF) [file pone.0171413.s003.pdf]

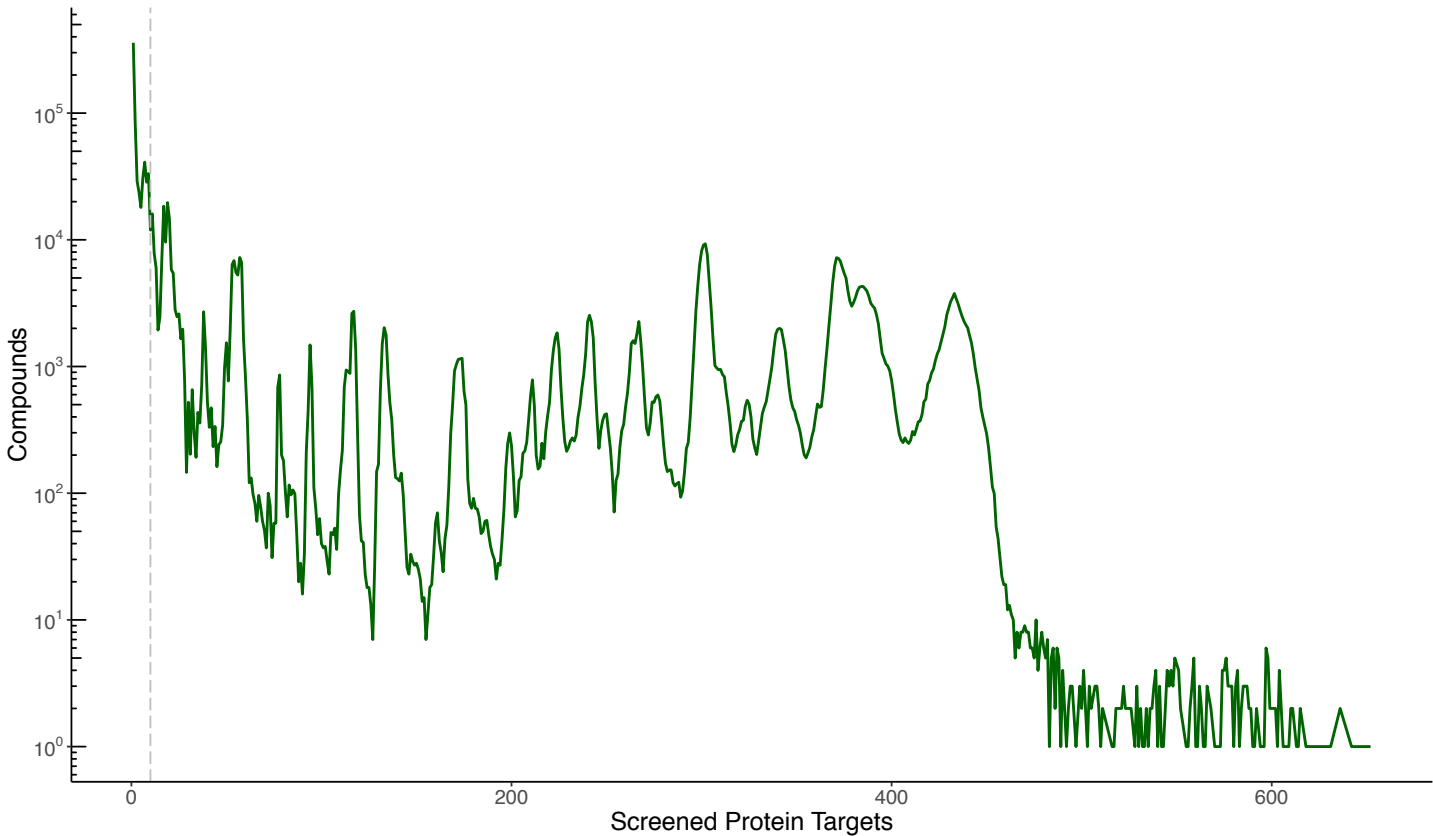

Supplement: S3 Fig — Data is included from all assay experiments in PubChem BioAssay annotated with one or more clearly defined protein targets, and reporting an active score for at least one small molecule. The dashed vertical line is drawn at 10 targets, which is the minimum value we categorize in this study as a “highly screened” compound. (PDF) [file pone.0171413.s004.pdf]

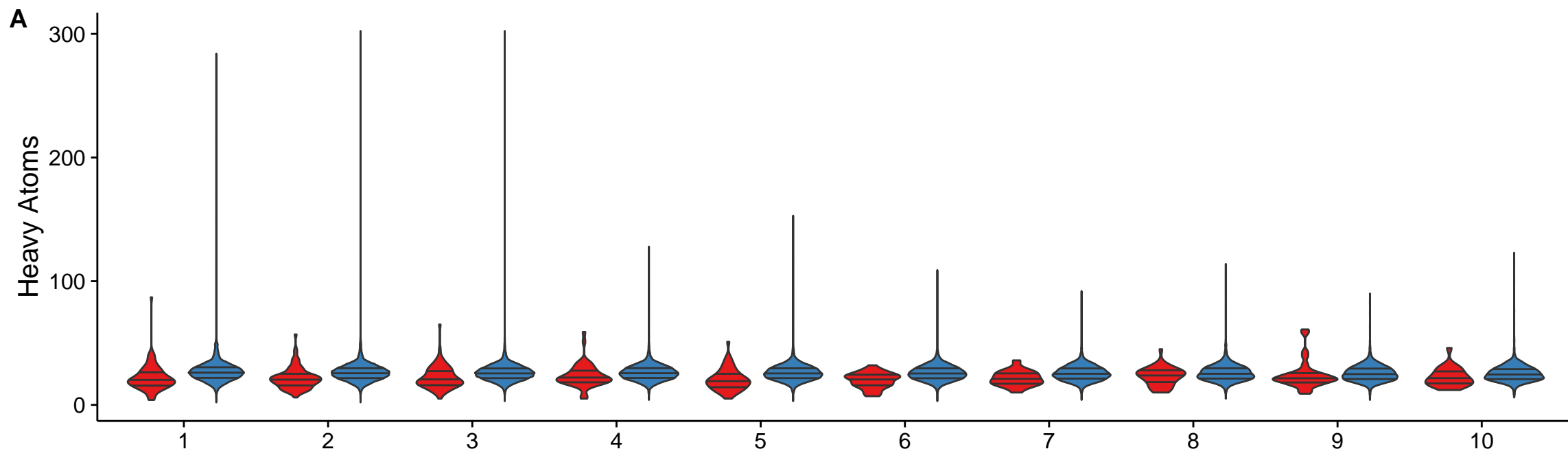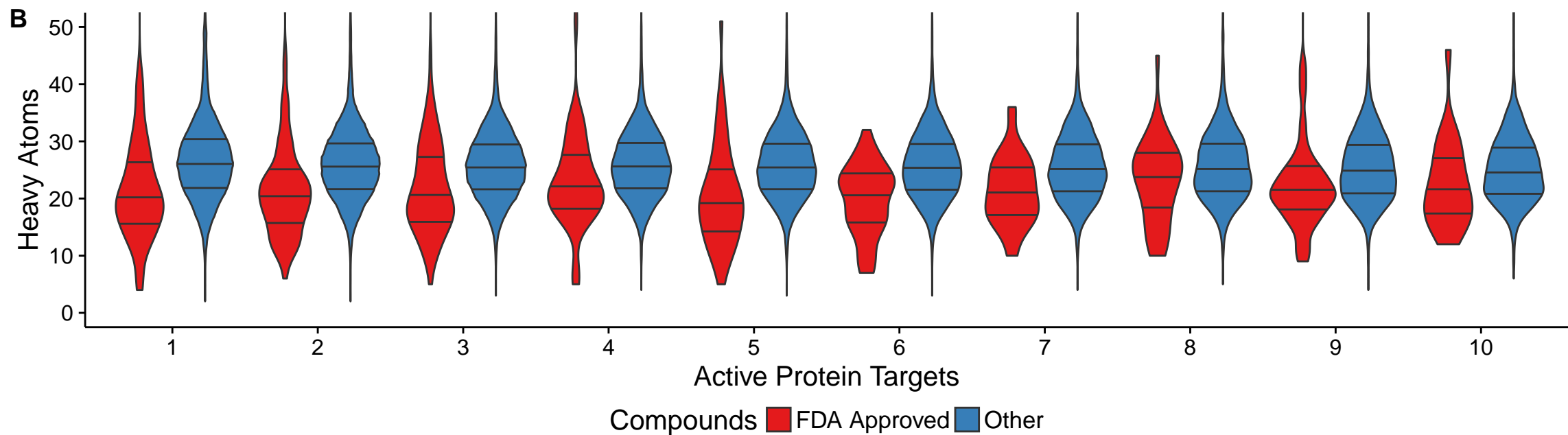

Supplement: S4 Fig — Violin plot with horizontal lines drawn at the 0.25, 0.5, 0.75 quantiles with tails trimmed to the range of data, as described in the “Target Selectivity by Molecular Size” section of S1 Text. Molecule size is quantified here by the number of non-hydrogen (heavy) atoms. (A) Target selectivity vs. molecular size across the entire range (y axis) of variation in these data. (B) Target selectivity vs. molecular size zoomed in on the y-axis to show more detail. (PDF) [file pone.0171413.s005.pdf]

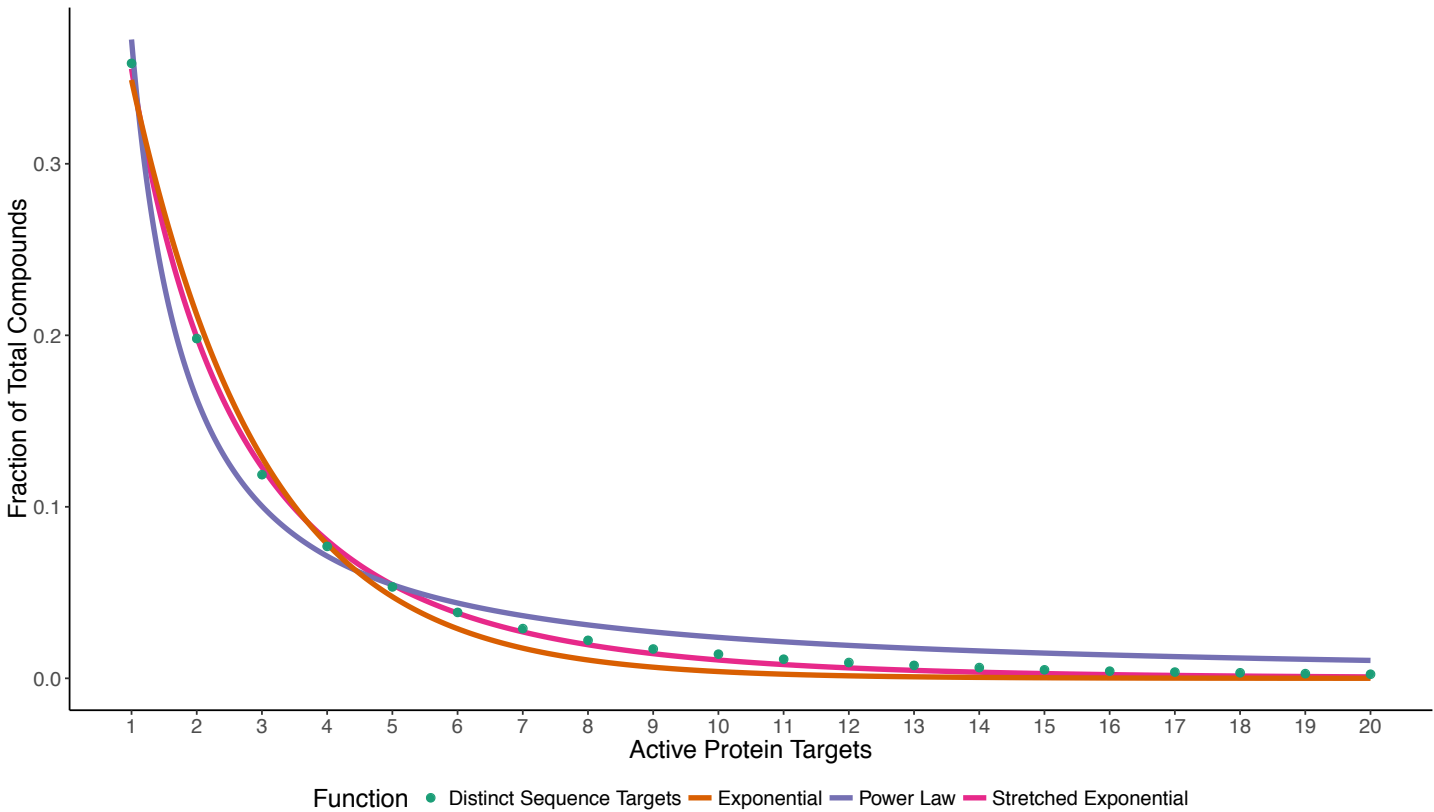

Supplement: S5 Fig — The distribution of cluster selectivity counts for non-FDA approved compounds as shown in Fig 4, along with best fit lines using two-parameter versions of the exponential, power law, and stretched exponential functions, as described in the “Stretched Exponential Selectivity Distribution” section of S1 Text. The stretched exponential fits the data better than the exponential, or power law functions (with R2 = 0.99912, 0.99131, and 0.97916 respectively). (PDF) [file pone.0171413.s006.pdf]

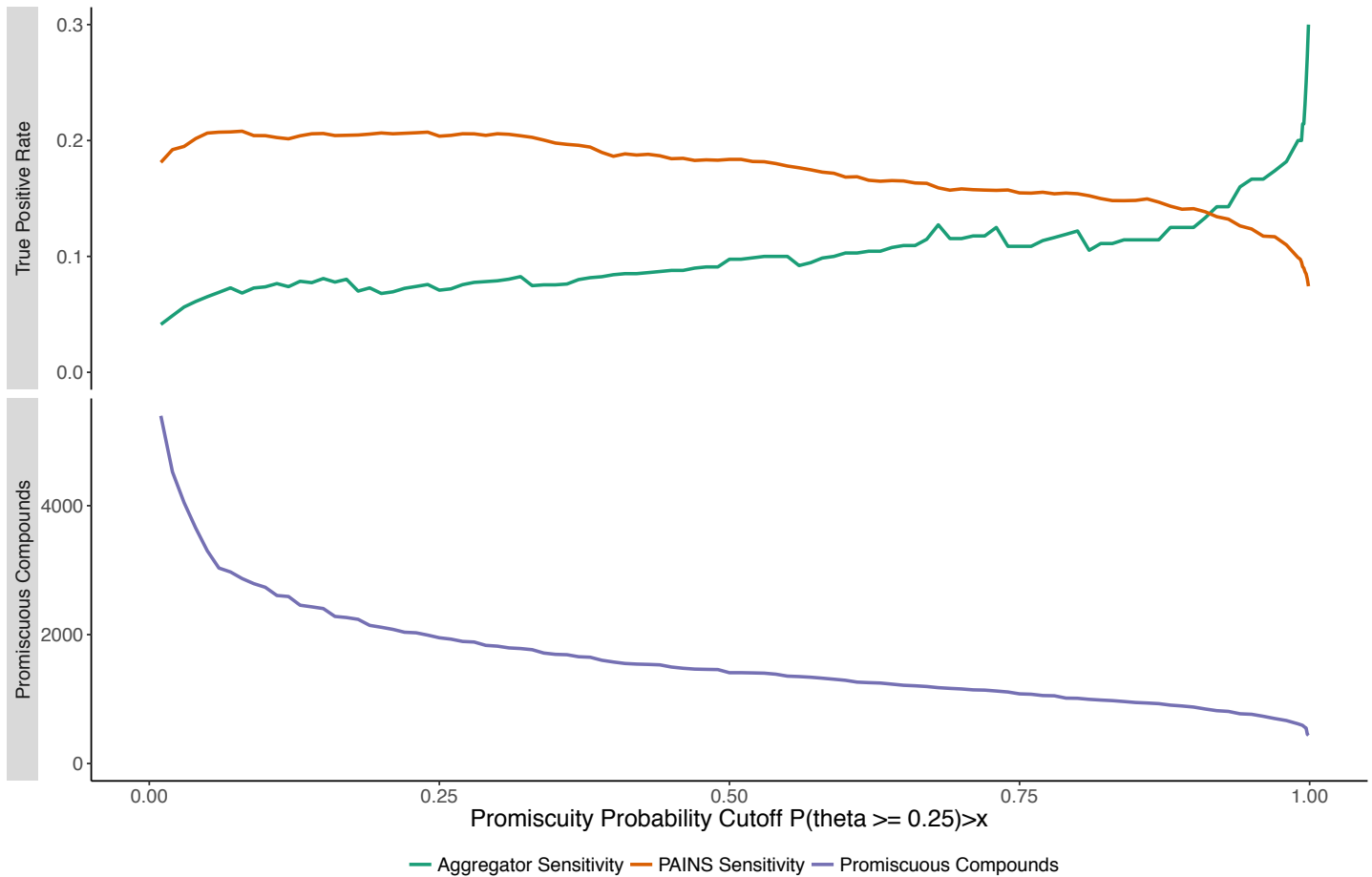

Supplement: S6 Fig — The top panel shows the sensitivity (true positive rate) of PAINS and aggregators to categorize promiscuous compounds throughout a range of promiscuity probability cutoffs P(θ ≥ 0.25) > x over the range x = [0.01, 0.9999]. The bottom panel shows the number of promiscuous compounds at each cutoff value. While the Bayesian model classifies all highly screened compounds, the values shown in the top panel are computed only with the subsets that were classified as aggregators/non-aggregators, and PAINS/non-PAINS respectively. There is an intersection of 44 compounds in this analysis which are classified as both PAINS and aggregators, out of 56330 highly screened active compounds which were tested and had valid results in both. (PDF) [file pone.0171413.s007.pdf]

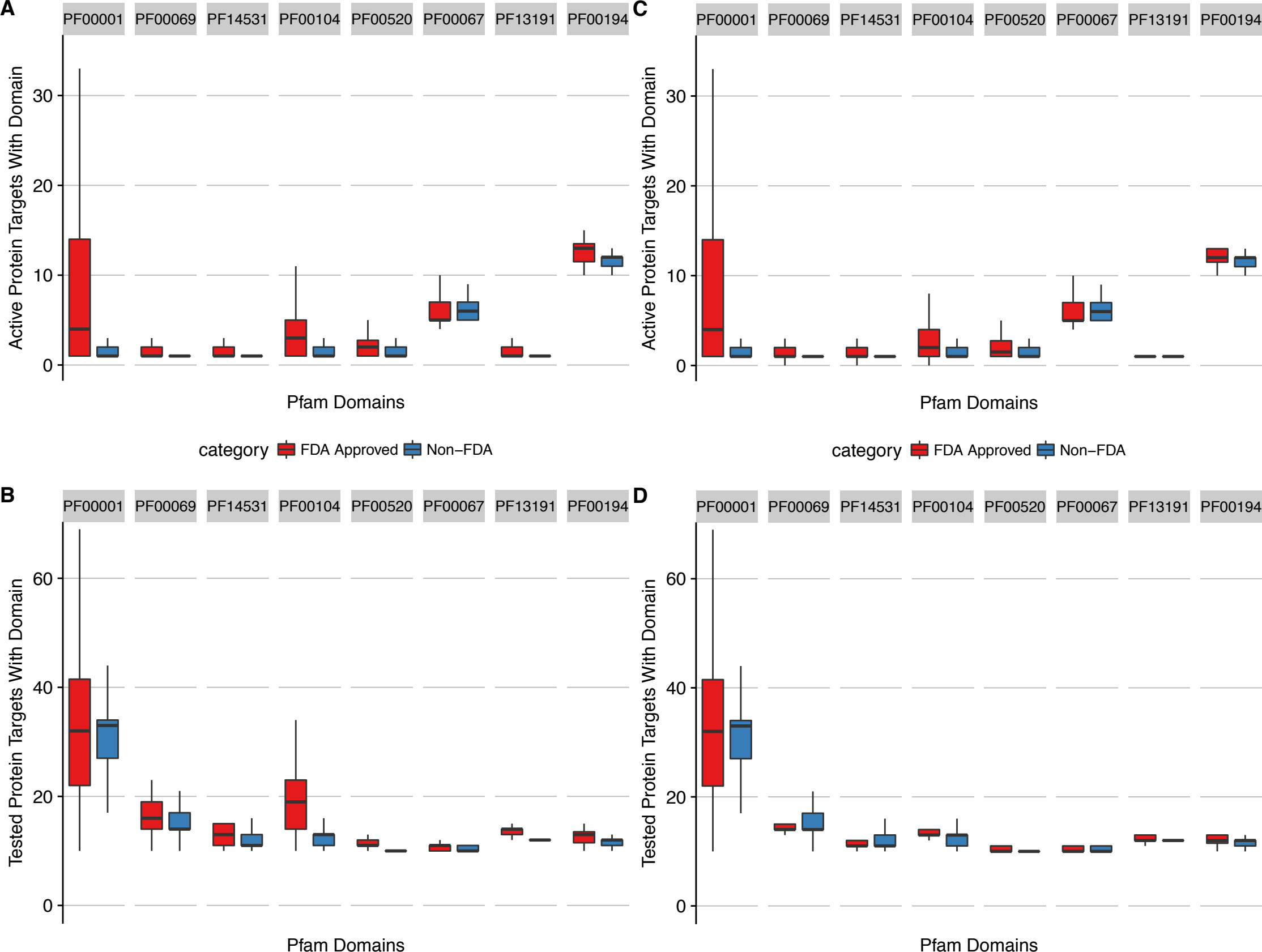

Supplement: S7 Fig — The distribution of active and tested targets for FDA approved and non-FDA approved compounds within targets sharing a common Pfam domain, as described in the “Target Selectivity Distribution Among Targets Sharing a Common Protein Domain” section of S1 Text. See Table V in S1 Text for the full names of each domain, as well as the number of FDA approved compounds, non-FDA compounds, and total protein targets for each domain. Horizontal lines are positioned at the 25%, 50%, and 75% quantiles for each distribution, with whiskers extending to 1.5 times the inter-quartile range. (A) The distribution of active protein targets within each domain. (B) The distribution of total screened targets within each domain. (C) The same as A except with iterative random removal of activity outcomes from the most highly screened FDA Approved drugs, such that the median number of screened targets for the FDA approved compounds is equal to or slightly less than that for non-FDA approved compounds, to enable cross-comparison without bias due to screening volume. (D) The same as B except with iterative random removal of activity outcomes from the most highly screened FDA Approved drugs, such that the median number of screened targets for the FDA approved compounds is equal to or slightly less than that for non-FDA approved compounds, to enable cross-comparison without bias due to screening volume. (PDF) [file pone.0171413.s008.pdf]
